# A semi-supervised adaptive Markov Gaussian embedding process (SAMGEP) for prediction of phenotype event times using the electronic health record

**DOI:** 10.1038/s41598-022-22585-3

**Published:** 2022-10-22

**Authors:** Yuri Ahuja, Jun Wen, Chuan Hong, Zongqi Xia, Sicong Huang, Tianxi Cai

**Affiliations:** 1 Department of Biostatistics, Harvard T.H. Chan School of Public Health, 677 Huntington Ave, Boston, MA 02115 USA; 2 Harvard Medical School, Boston, MA USA; 3 Department of Medicine, NYU Langone Health, New York, NY USA; 4 Department of Neurology, University of Pittsburgh, Pittsburgh, PA USA; 5 Division of Rheumatology, Inflammation, and Immunity, Brigham and Women’s Hospital, Boston, MA USA; 6 VA Boston Healthcare System, Boston, MA USA

**Keywords:** Data processing, Machine learning, Statistical methods, Information technology, Statistics

## Abstract

While there exist numerous methods to identify binary phenotypes (i.e. COPD) using electronic health record (EHR) data, few exist to ascertain the timings of phenotype events (i.e. COPD onset or exacerbations). Estimating event times could enable more powerful use of EHR data for longitudinal risk modeling, including survival analysis. Here we introduce Semi-supervised Adaptive Markov Gaussian Embedding Process (SAMGEP), a semi-supervised machine learning algorithm to estimate phenotype event times using EHR data with limited observed labels, which require resource-intensive chart review to obtain. SAMGEP models latent phenotype states as a binary Markov process, and it employs an adaptive weighting strategy to map timestamped EHR features to an embedding function that it models as a state-dependent Gaussian process. SAMGEP’s feature weighting achieves meaningful feature selection, and its predictions significantly improve AUCs and F1 scores over existing approaches in diverse simulations and real-world settings. It is particularly adept at predicting cumulative risk and event counting process functions, and is robust to diverse generative model parameters. Moreover, it achieves high accuracy with few (50–100) labels, efficiently leveraging unlabeled EHR data to maximize information gain from costly-to-obtain event time labels. SAMGEP can be used to estimate accurate phenotype state functions for risk modeling research.

## Introduction

Electronic Health Record (EHR) data collected during the routine delivery of care have in recent years enabled countless opportunities for translational and clinical research^1–3^. Comprising freeform clinical notes, lab results, prescriptions, and codified features including International Classification of Diseases (ICD) and Current Procedural Terminology (CPT) billing codes, EHRs encode rich information for research. However, EHRs’ lack of gold-standard phenotype labels limits utilization of these data to precisely estimate epidemiological parameters such as prevalence and treatment effects, or to develop and validate well-calibrated risk prediction models for clinical events. Phenotype surrogate features such as ICD diagnosis codes often exhibit dismal specificity that can bias or de-power the downstream study^4,5^. Meanwhile, manual annotation of phenotypes via chart review is laborious and unscalable. These limitations become even more pronounced when the object of interest is the *timing* of clinical events, which is important for survival analysis or evaluating disease course. Event time surrogates derived from EHR codes often exhibit systematic biases, and multiple features may be needed to accurately predict timing^6–8^.

For binary phenotypes, researchers have proposed a variety of unsupervised and semi-supervised methods requiring few-to-no manually-annotated gold-standard labels^9–20^. However, few methods exist to predict phenotype event times. Chubak et al. developed a rule-based algorithm that predicts breast cancer recurrence time based on the earliest encounter times of expert-specified codes^8^. Hassett et al. proposed a similar algorithm averaging the peak times of selected codes^7^. Uno et al. expanded on this by using points of maximal increase in lieu of peak values, and adjusting for systematic temporal biases between code timings and phenotype onset^6^. While these approaches achieve notable performance, they are limited by (1) reliance on a limited, curated set of predictive codes, and (2) sensitivity to sparsity, a common characteristic of EHR data. In addition, these algorithms cannot identify multiple event times as might be pertinent for a relapsing and remitting phenotype such as multiple sclerosis.

Using machine learning to predict event times can potentially address these limitations. Traditional supervised learning methods such as logistic regression, random forest, and naive Bayes are suboptimal for modeling longitudinal processes as they cannot account for intertemporal associations in either outcomes or features. Recurrent neural networks (RNNs), designed for sequence data and well-conditioned to high feature dimensions, have had widespread prediction applications using longitudinal data^21–25^. One recent RNN-based method optimized for healthcare data, the Reverse Time Attention Model, (RETAIN), offers particularly notable accuracy to this end^26^. However, neural networks often require large numbers of training labels to achieve stable performance, which can be very expensive to attain. Consequently, existing applications of RNNs to EHR-based prediction typically use readily available outcome measures such as discharge billing codes, hindering application to outcomes without reliable codified proxies. Additionally, apart from RETAIN these models are generally not intuitively interpretable.

On the other end of the spectrum, researchers have developed unsupervised computational models of chronic disease progression that do not use any gold-standard labels^26–31^. Many of these approaches employ Hidden Markov Models (HMMs) in which latent states represent disease stages or status. For instance, Jackson et al. apply a multistage discrete HMM to aneurysm screening, Sukkar et al. apply one to Alzheimer’s disease, and Wang et al. apply a continuous HMM to progression of chronic obstructive pulmonary disease^26–29^. However, the latent states learned from these unsupervised models may not be reflective of the target phenotype or even clinically relevant.

In this paper we propose Semi-supervised Adaptive Markov Gaussian Embedding Process (SAMGEP), a label-efficient, semi-supervised machine learning method to predict the presence of a well-defined binary phenotype over time using longitudinal EHR data. By leveraging large-scale unlabeled data to train the temporal prediction model, SAMGEP makes efficient use of limited (~ 50–100) gold-standard event labels. Unlike existing event identification algorithms, SAMGEP can leverage hundreds of sparse EHR features rather than a handful of surrogates by combining features and their embeddings into dense patient-timepoint embeddings via a novel data-driven weighting procedure. It then models the patient-timepoint embedding progression as a Gaussian Process emission of an HMM to predict the target phenotype, combining desirable aspects of existing phenotyping methods.

## Results

### Model overview

**Figure 1:**
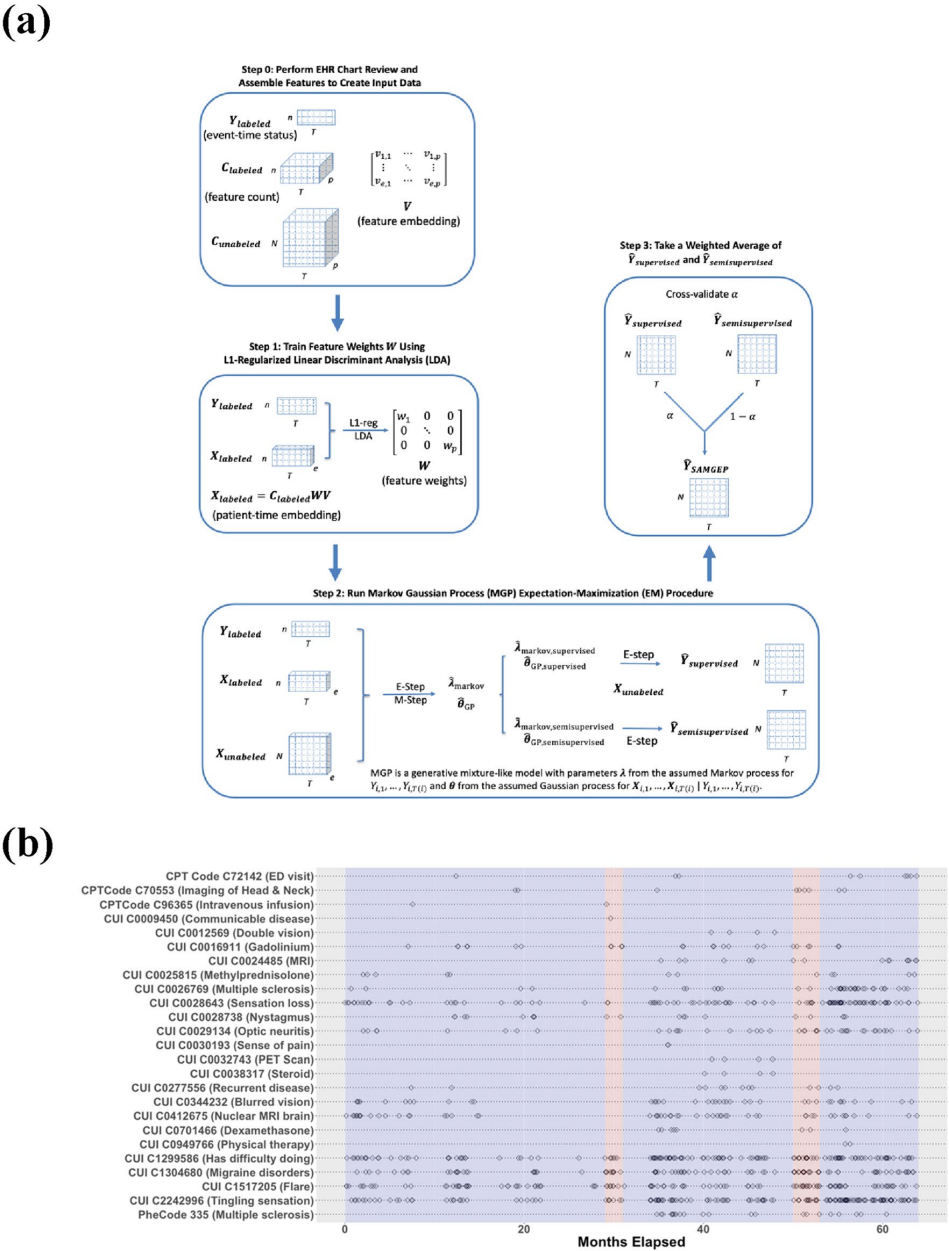
(**a**) Schematic of the overall SAMGEP algorithm. (**b**) Depiction of the sparsity and temporal irregularity of EHR data. In this study we aim to predict multiple sclerosis (MS) relapse event times (red bands) using timestamped EHR feature observations (black diamonds).

SAMGEP predicts well-defined (i.e. not new) binary phenotype processes from longitudinal EHR data with few observed gold-standard event labels. It does so in four steps: (i) assembling time-dependent candidate features, (ii) optimizing weights for combining feature embeddings into dense patient-timepoint embeddings, (iii) fitting supervised and semi-supervised Markov Gaussian Process (MGP) models to the embedding process to predict phenotype status over time, and (iv) taking a weighted average of these semi-supervised and supervised predictions with weights determined adaptively to optimize prediction performance. Figure 1A illustrates the overarching SAMGEP procedure, and Fig. 1B depicts the form of raw longitudinal EHR data for input into SAMGEP. A detailed description of the SAMGEP procedure is included in the “Methods”. R source code is available at https://cran.r-project.org/web/packages/SAMGEP/index.html.

### Feature selection

**Figure 2:**
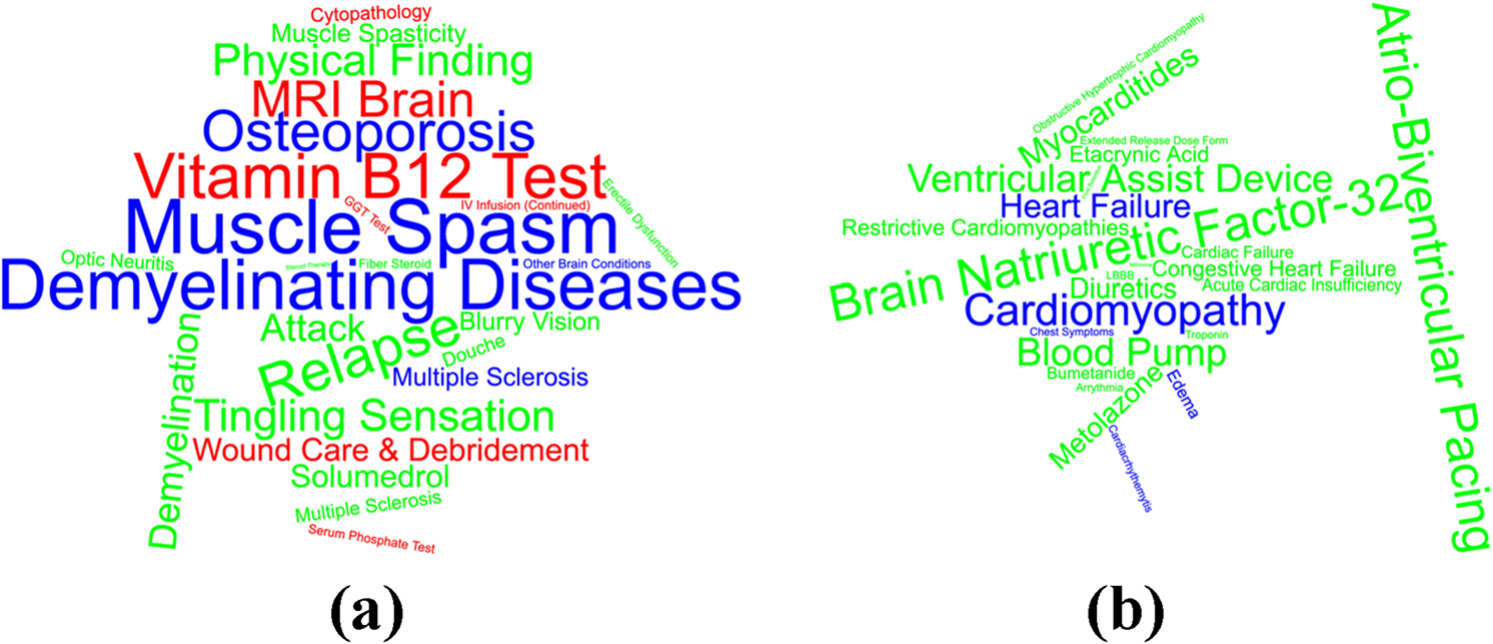
Feature word clouds for (**a**) MS relapse and (**b**) HF onset using the product of SAMGEP’s feature weights and the empirical standard deviations of corresponding features. See the “Evaluation metrics” subsection of the “Methods” for details.

Figure 2 depicts feature clouds generated using SAMGEP’s feature weights for identification of (A) MS relapse and (B) HF onset. SAMGEP identified PheCodes for demyelinating diseases and muscle spasm, CPT codes for vitamin B12 testing and MRI brain, and CUIs for “relapse” and “tingling sensation” as most predictive of MS relapse. For identification of HF onset, SAMGEP selected PheCodes for heart failure and cardiomyopathy, and CUIs for “brain natriuretic factor 32” and “atrio-biventricular pacing.” In both cases, SAMGEP reassuringly selected and upweighted appropriate, clincially relevant features for the respective outcomes.

### Robustness to data generative characteristics

**Figure 3:**
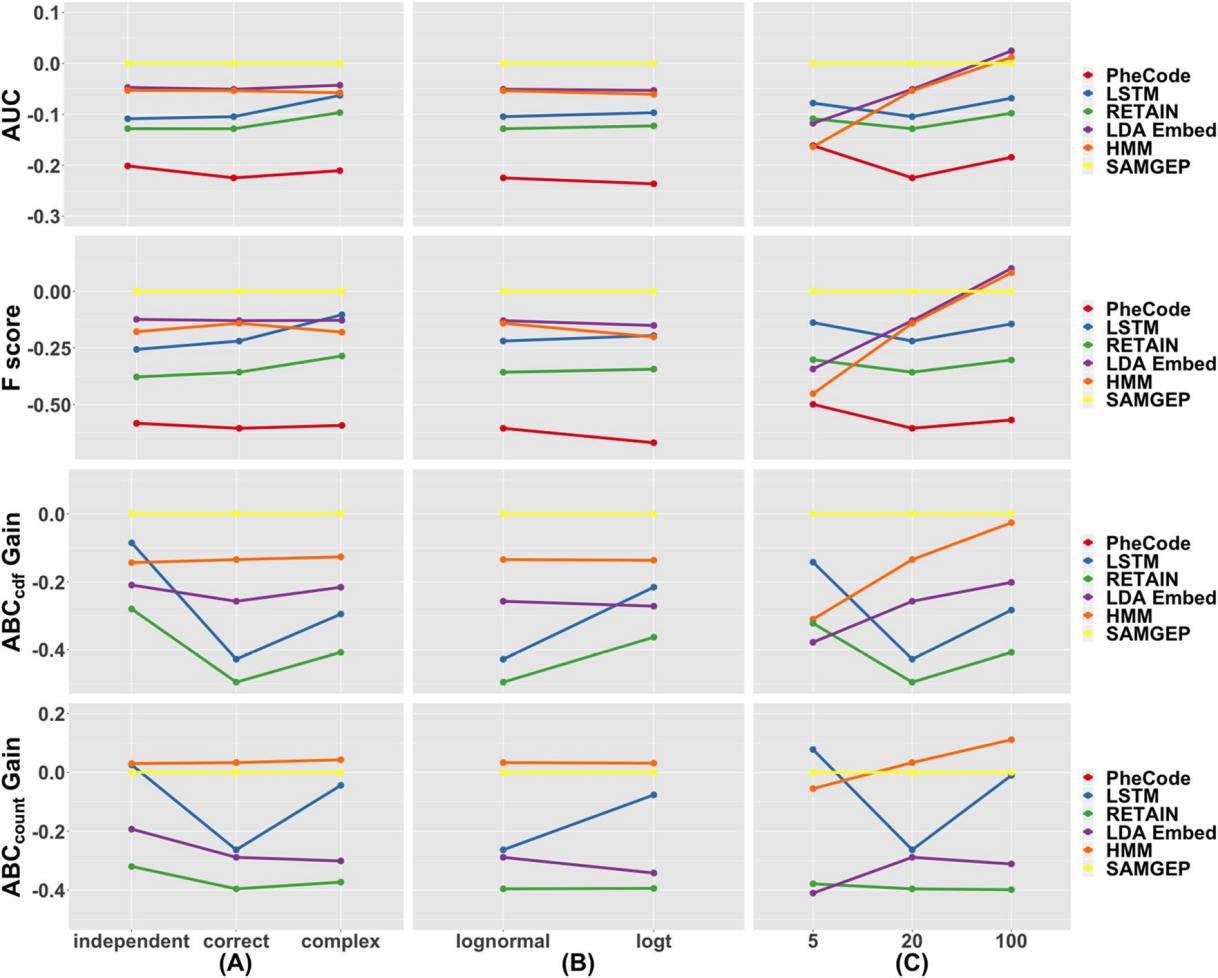
Robustness of SAMGEP and comparator methods’ AUCs, F scores, ABC_cdf_ gains, and ABC_count_ gains to various generative parameters, including the (**a**) specification of $Y|T$, (**b**) specification of ${\varvec{C}}|Y$, and (**c**) number of informative (i.e. non-sparse) features. Details of the experiments are delineated in the “Simulation study” subsection of the “Methods”, and more extensive results are displayed in Supplementary Fig. S5.

Figure 3 explores SAMGEP and comparators’ robustness to various generative model specifications. Note that only relative performance between methods, not absolute performance, is meaningful as different generative settings may portend disparate inherent levels of information; nevertheless, we also include absolute performance metrics in Supplementary Table S3.

Panels A and B demonstrate unsurprisingly that SAMGEP outperforms RETAIN and LSTM when SAMGEP’s distributional assumptions regarding (A) ${\varvec{Y}}|{\varvec{T}}$ and (B) ${\varvec{X}}|{\varvec{Y}}$ are correctly specified. That said, SAMGEP achieves strong relative performance notwithstanding misspecification of ${\varvec{Y}}|{\varvec{T}}$ or ${\varvec{X}}|{\varvec{Y}}$. Practically this indicates that SAMGEP is robust to model misspecification, though the more the true distribution diverges from SAMGEP’s assumption, the less desirable SAMGEP is relative to highly flexible deep learning models.

Panel C demonstrates that SAMGEP provides more benefit over LDA_Embed_ and HMM the more sparsely information is distributed over features (i.e. 5 rather than 100 informative features out of 150), reflecting the robustness of SAMGEP’s L_1_-regularized weighting protocol to information sparsity. This robustness makes SAMGEP well-conditioned for the EHR, which typically contains a handful of informative features out of millions. Meanwhile, SAMGEP offers the most benefit over LSTM and RETAIN, which also utilize L_1_ weighting, when the number of informative features is 20. This is likely due to the fact that we used embeddings of dimension $m=10$, resulting in bias when the true number of informative dimensions exceeds 10.

### Identification of MS relapse and HF onset using real-world EHR data

**Figure 4:**
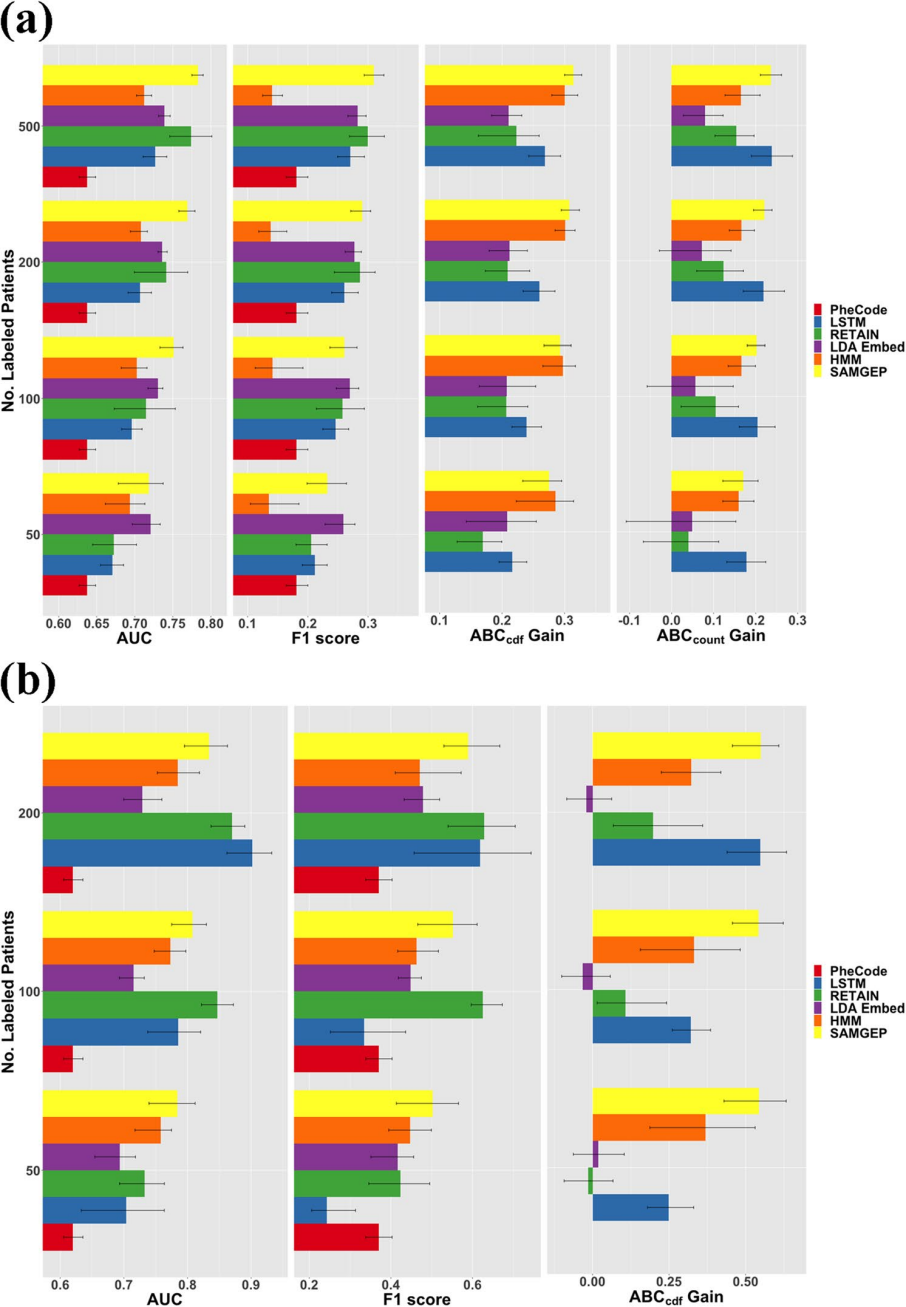
Predictive accuracies of SAMGEP and various comparator methods using real-world EHR data to predict (**a**) MS relapse with $n\in \{\mathrm{50,100,200,500}\}$ labeled patients, and (**b**) HF onset with $n\in \{\mathrm{50,100,200}\}$ labels. 95% confidence intervals were empirically estimated by bootstrapping with 100 replicates. See the “Evaluation metrics” subsection of the “Methods” for details about the evaluation metrics. More extensive results are displayed in Supplementary Fig. S6.

Figure 4 depicts mean AUCs, F1 scores, ABC_cdf_ gains, and ABC_count_ gains for SAMGEP and key comparator methods predicting (A) MS relapse and (B) HF onset using real-world EHR data. A plot with all comparators is included in Supplementary Fig. S6, and a line plot over the number of observed samples $n$ is included in Supplementary Fig. S8. For identification of MS relapse, SAMGEP achieved significantly higher AUCs than all other methods, though RETAIN approached SAMGEP for $n=500$ labels. SAMGEP also achieved F1 scores equivalent to that of the top-performing method for all $n$. LSTM and RETAIN achieved lackluster AUCs for $n\in \{50$,$100$,$200\}$, an unsurprising result given that deep learning models are well known to exhibit unstable performance in small data settings. SAMGEP also achieved the highest ABC_cdf_ gains, though not significantly so relative to HMM. Finally, SAMGEP achieved the highest ABC_count_ gains, though statistically equivalent to LSTM per Student’s t test and only marginally superior to HMM. The fact that SAMGEP, HMM, and LSTM were the top performers by both ABC metrics, despite HMM and LSTM’s unremarkable AUCs, suggests that jointly modeling $\{{Y}_{i,1},\dots ,{Y}_{i,T\left(i\right)}\}$ is singularly beneficial for longitudinal phenotype process prediction. LDA_embed_ does not even significantly improve upon the null model’s ABC metrics for $n\in \{\mathrm{50,100,200}\}$, demonstrating that accurately predicting phenotype states at individual timepoints does not necessarily translate into accurate phenotype process prediction.

For identification of HF onset, SAMGEP achieved the highest AUCs and F1 scores for $n=50$ but was outperformed by RETAIN for $n=100$, and both RETAIN and LSTM for $n=200$. SAMGEP achieved the highest ABC_cdf_ gains across the board but was statistically equivalent to HMM for $n=50$ and LSTM for $n=200$. The fact that SAMGEP, LSTM, RETAIN, and HMM achieved high accuracies across metrics reflects the powerful benefit of leveraging the full time sequence when predicting the onset of a chronic disease, wherein ${Y}_{t} | \left({Y}_{t-1}=1\right)=1 \mathrm{with probability }1.$ Moreover, the fact that RETAIN often outperformed SAMGEP for HF onset but not MS relapse identification suggests that SAMGEP offers particular benefit over deep learning for prediction of a relapsing and remitting process, which expends more degrees of freedom than prediction of disease onset. Notably, for both prediction tasks SAMGEP is robust to low $n$, whereas the deep learning benchmarks require larger labeled sets to achieve sufficient performance stability.

While SAMGEP does not always outperform all comparators, its consistency is notable. Whereas some comparators achieve high accuracy by only certain metrics or on only one of our two outcomes, SAMGEP achieves consistently strong performance, and for $n=50$ it always achieves statistically equivalent accuracy to the top performing method. It demonstrates proficiency at predicting both phenotype states at individual timepoints and phenotype processes over time, most notably in the label-poor setting (i.e. 50–100 labels). Finally, it achieves high accuracy on two contrasting phenotypic outcomes, bolstering our claim of generalizability.

### Estimation of cumulative probability and counting process curves

**Figure 5:**
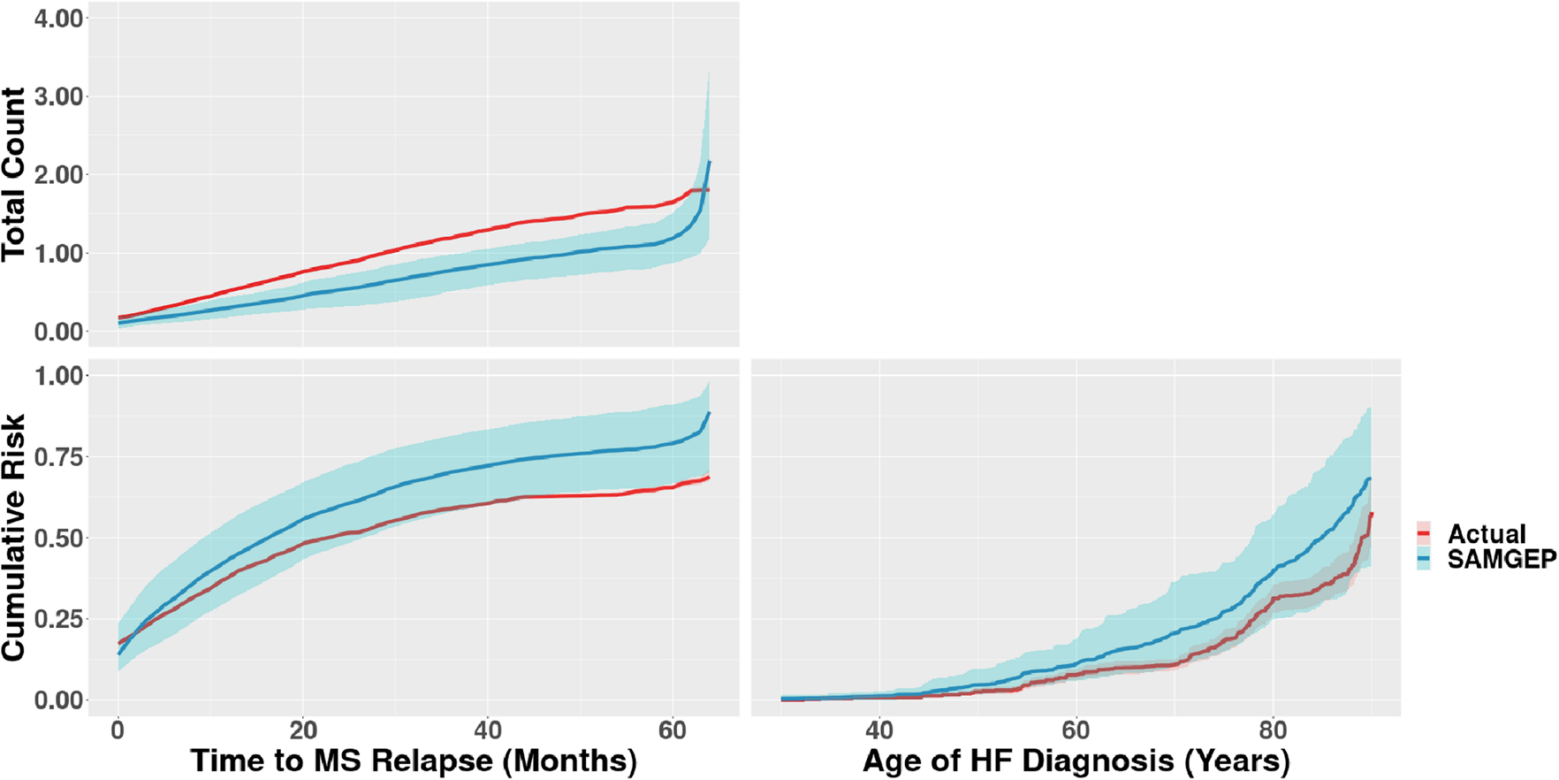
Estimation of population-wide cumulative probability (bottom) and counting process (top) curves for MS relapse (left) and HF development (right) using the predictions of SAMGEP trained with $n=100$ labeled patients. “Actual” curves were estimated using all available labels. Only labeled sets were used to generate curve estimates in order to enable unbiased comparison between the SAMGEP-predicted and actual curves. 95% confidence intervals were empirically estimated by bootstrapping with 100 replicates. More extensive results are displayed in Supplementary Fig. S7.

Figure 5 depicts the estimated CDF, obtained as $\widehat{F}\left(t\right)={N}^{-1}{\sum }_{i=1}^{N}{\widehat{F}}_{i,t}$, and counting process, obtained as $\widehat{N}\left(t\right)={N}^{-1}{\sum }_{i=1}^{N}{\widehat{N}}_{i,t}$, based on the identifications of SAMGEP using $n=100$ labels, along with 95% confidence intervals. Notably, CDF estimation using SAMGEP’s identifications is relatively unbiased for onset of both (A) first MS relapse and (B) HF. As Supplementary Fig. S7 demonstrates, comparator methods’ predictions tends to markedly overestimate the true cumulative risk, whereas SAMGEP consistently achieves the least biased estimates.

Counting process estimation using SAMGEP’s MS relapse identifications appears to systematically but slightly underestimate the true counting process. SAMGEP significantly improves upon comparators later in patients’ disease courses, where all other methods except HMM appear to markedly overestimate. SAMGEP’s identifications again improve bias at the expense of increased variance, overall significantly improving ABC_count_.

## Discussion

While identification of binary phenotypes using EHR data is well-trodden in the literature, identification of longitudinal phenotype processes, or event times, remains underdeveloped. As our results demonstrate, accurate identification of a patient’s phenotype status overall—or even at a particular timepoint—does not necessarily translate into accurate phenotype process prediction. SAMGEP accurately predicts phenotype processes, constituting a meaningful step forward for computational phenotyping.

SAMGEP excels relative to existing methods because it (1) can leverage numerous EHR features, which is particularly important for phenotypes that are insufficiently represented by a handful of surrogates (i.e. ICD codes); (2) incorporates prior knowledge by utilizing low-dimensional feature embeddings trained using all available EHR data; (3) can efficiently utilize few gold-standard labels by leveraging unlabeled data in a semi-supervised manner; and (4) jointly models the time sequence of relapses and features rather than treating individual timepoints as independent observations. Figures 4A, 5, and Supplementary Fig. S7 demonstrate that SAMGEP achieves particularly accurate (per ABC_cdf_ and ABC_count_) and unbiased CDF and counting process estimates for a relapsing-and-remitting phenotype, particularly in the setting of few (i.e. 50–100) observed labels. Manual annotation of event times is an extremely laborious process, so SAMGEP’s label efficiency is a key attribute. Conversely, popular deep learning models such as RETAIN and LSTM are well known to exhibit low performance stability in such small data settings.

We envision SAMGEP’s phenotype process predictions being used as outcomes in a downstream clinical or epidemiological study. For instance, researchers aiming to measure the effect of an MS treatment on the rate of relapse could (1) annotate the relapse histories of ~ 100 patients via chart review, (2) use SAMGEP to estimate cumulative relapse probabilities for all remaining patients, and (3) use these relapse probabilities as outcomes to measure treatment effect. Further research is warranted to assess the bias-variance tradeoff of such a workflow relative to traditional predictive modeling methods using the labeled set alone.

While SAMGEP is label-efficient, manually annotating even 50–100 event time labels is a labor-intensive process. Modifying SAMGEP to handle current status labels—indicators of phenotype status at censor time—would greatly diminish the chart review time required to utilize the algorithm for phenotype onset prediction. Further work is warranted to explore this possibility.

Finally, SAMGEP in its current form is not robust to covariate shift between the labeled and unlabeled sets—a realistic scenario in cases where labeled patients are not a random subsample of the study population. Inverse probability weighting could be employed to eliminate this sampling bias, and we leave this as a future study direction.

In summary, SAMGEP is a novel semi-supervised machine learning method that accurately predicts the course of a binary phenotype over time using EHR data with few observed event time labels. Singularly adept at estimating cumulative probability and counting process functions, SAMGEP promises to enable more powerful use of EHR data for epidemological research involving event timings, including survival analysis.

## Methods

### Assembling predictive features

To assemble feature counts and define phenotype states, we consider consecutive non-overlapping time periods starting at the patient’s first ICD code for the target phenotype. Candidate features include log-transformed counts of ICD codes, RxNorm drug codes, CPT codes, lab tests, and mentions of clinical concepts in a patient’s record. Features can be selected manually or identified automatically via label-free methods such as surrogate-assisted feature extraction^33^. Fig. 1B depicts the form of raw EHR feature data for MS relapse identification, with red bands indicating relapse events. Since SAMGEP employs sparse feature weighting to select informative features, it is preferable to include features liberally rather than aiming for parsinominousness in feature assembly.

Henceforth we let ${{\varvec{V}}}_{m\times p}$ denote the matrix of *m*-dimensional embedding vectors for *p* features. See the Producing Feature Embeddings section of the Supplementary Materials for details on how ${{\varvec{V}}}_{m\times p}$ is pre-trained. We use $i$, $j$, and $t$ to index patients, raw features, and time periods respectively. We assume there are $N$ patients and ${T}_{i}$ periods for patient $i$ in our dataset. For patient $i$ at timepoint $t$, let ${{\varvec{C}}}_{{\varvec{i}},{\varvec{t}}}$ denote the $p$-dimensional raw feature vector and ${Y}_{i,t}\in \{\mathrm{0,1}\}$ denote the phenotype state. Let ${H}_{i}$ = log(mean healthcare encounter count per month + 1) in patient $i$’s record, a measure of healthcare utilization. Finally, we assume that ${{\varvec{Y}}}_{{\varvec{i}}}=({Y}_{i,1},\dots ,{Y}_{i,{T}_{i}})$ is annotated on a limited set of $n\ll N$ patients, but ${{\varvec{C}}}_{{\varvec{i}}}=\left({{\varvec{C}}}_{{\varvec{i}},1},\dots ,{{\varvec{C}}}_{{\varvec{i}},{{\varvec{T}}}_{{\varvec{i}}}}\right)$ is observed for all $N$ patients.

### Producing patient-timepoint embeddings

SAMGEP leverages pre-trained feature embeddings ${\varvec{V}}$ to compress the high-dimensional feature vector ${{\varvec{C}}}_{{\varvec{i}},{\varvec{t}}}$ to a low-dimensional patient-timepoint embedding ${{\varvec{X}}}_{{\varvec{i}},{\varvec{t}}}$**,** which can be efficiently modeled as a Gaussian process. We compute ${{\varvec{X}}}_{{\varvec{i}},{\varvec{t}}}$ as a weighted sum over feature embeddings:

1 $${{\varvec{X}}}_{{\varvec{i}},{\varvec{t}}}={{\varvec{C}}}_{{\varvec{i}},{\varvec{t}}}{\varvec{W}}{\varvec{V}},\mathrm{ for }i=1,\dots , N \mathrm{and }t=1, \dots , {T}_{i},$$

where $${\varvec{W}}=\left(\begin{array}{ccc}{W}_{1}& \cdots & 0\\ \vdots & \ddots & \vdots \\ 0& \cdots & {W}_{p}\end{array}\right)$$**,** ${W}_{j}$ is the unknown weight for the *j*th feature, and ${W}_{1}=1$ to ensure identifiability. We choose ${\varvec{W}}$ via L1-regularized linear discriminant analysis maximizing.

$${\varvec{D}}\left({\varvec{W}}\right)={\left({{\varvec{\mu}}}_{1}-{{\varvec{\mu}}}_{0}\right)}^{{\varvec{T}}}{{\varvec{\Sigma}}}_{{\varvec{X}}}^{-1}\left({{\varvec{\mu}}}_{1}-{{\varvec{\mu}}}_{0}\right)-\lambda {\left|\left|{\varvec{W}}\right|\right|}_{1}^{1},$$

where ${\Vert {\varvec{W}}\Vert }_{1}^{1}$ denotes the L_1_ norm of ${\varvec{W}}$, $\lambda \ge 0$ is the tuning parameter,

$${{\varvec{\mu}}}_{{\varvec{y}}}=\frac{{\sum }_{i=1}^{N}{\sum }_{t=1}^{{T}_{i}}{{\varvec{X}}}_{{\varvec{i}},{\varvec{t}}}{I(Y}_{i,t}=y)}{{\sum }_{i=1}^{N}{\sum }_{t=1}^{{T}_{i}}{I(Y}_{i,t}=y)} , \mathrm{and} {{\varvec{\Sigma}}}_{\mathbf{X}}=\frac{1}{{\sum }_{i=1}^{N}{T}_{i}}{\sum }_{i=1}^{N}{\sum }_{t=1}^{{T}_{i}}({{{\varvec{X}}}_{{\varvec{i}},{\varvec{t}}}-{{\varvec{\mu}}}_{{{\varvec{Y}}}_{{\varvec{i}},{\varvec{t}}}})\left({{\varvec{X}}}_{{\varvec{i}},{\varvec{t}}}-{{\varvec{\mu}}}_{{{\varvec{Y}}}_{{\varvec{i}},{\varvec{t}}}}\right)}^{\boldsymbol{^{\prime}}}; y=\mathrm{0,1}.$$

We choose L_1_ regularization over L_2_ or other L_p_ to impose sparsity given that most input features are likely uninformative. We optimize ${\varvec{W}}$ using projected gradient ascent, where without loss of generality we assume that the first feature is a known highly predictive feature. The step-size at each iteration of ascent is chosen by line search, and $\lambda$ is optimized using fivefold cross-validation maximizing ${\varvec{D}}\left({\varvec{W}}\right)$ within the labeled set. In this study, we use ${\varvec{V}}$ generated via a singular value decomposition (SVD) procedure, so our process of inferring ${\varvec{X}}$ is similar in spirit to matrix factorization. That said, our embedding procedure does not account for inter-temporal information, so it departs from modern tensor factorization methods in that regard.

### Fitting MGP

MGP is a generative mixture-like model that combines two assumptions: (1) ${{\varvec{Y}}}_{{\varvec{i}}}$ follows a discrete time Markov process, and (2) ${{\varvec{X}}}_{{\varvec{i}}}=\left({{\varvec{X}}}_{{\varvec{i}},1},\dots ,{{\varvec{X}}}_{{\varvec{i}},{{\varvec{T}}}_{{\varvec{i}}}}\right)|{{\varvec{Y}}}_{{\varvec{i}}}$ follows a Gaussian process. This generative framework primes SAMGEP for the semi-supervised setting.

#### Discrete time Markov process assumption

We assume a Markov process model for ${{\varvec{Y}}}_{{\varvec{i}}}|{H}_{i}$ such that ${P(Y}_{i,t}=y\left|{Y}_{i,1},\dots ,{Y}_{i,t-1}, {H}_{i}\right)={P(Y}_{i,t}=y\left|{Y}_{i,t-1}{,H}_{i}\right)$. This model is specified by two rules:

2 $${P(Y}_{i,1}=1\left|{H}_{i}\right)={\pi }_{init}\left({H}_{i}\right); {P(Y}_{i,t}=y\left|{Y}_{i,t-1}={y}_{t-1},{H}_{i}\right)\equiv {\pi }_{t}\left({y}_{t-1},{H}_{i}\right) \mathrm{for} t>1,$$

where {${\pi }_{init},{\pi }_{t}\left({y}_{t-1}\right) \forall t>1\}$ are unknown transition probabilities that fully specify the Markov model. We further assume that for some parameters ${{\varvec{\lambda}}}_{\mathrm{markov}}=\{{\lambda }_{init},{\lambda }_{0},{\lambda }_{1},{{\lambda }_{2},{\lambda }_{3},\lambda }_{H0},{\lambda }_{H}\}$,

$${\pi }_{init}({H}_{i})={expit(\lambda }_{init}+{\lambda }_{H0}{H}_{i}),\mathrm{ and}$$

$${\pi }_{t}\left({y}_{t-1}|{H}_{i}\right)=expit\left({\lambda }_{0}\left(1-{y}_{t-1}\right)+{\lambda }_{1}{y}_{t-1}+{{\lambda }_{2}t+{\lambda }_{3}\mathrm{log}t+ \lambda }_{H}{H}_{i}\right),$$

where $expit\left(x\right)=\mathrm{exp}\left(x\right)/[1+\mathrm{exp}\left(x\right)].$ We include both linear and log-linear time effects on ${\pi }_{t}({y}_{t-1}|{H}_{i})$ to better capture the temporal risk function without overfitting.

#### Gaussian process assumption

We assume the dense representations of patients’ EHRs (i.e. patient embeddings) over time follow a Gaussian process:

$${{\varvec{X}}}_{{\varvec{i}}}|{{\varvec{Y}}}_{{\varvec{i}}} \sim GP\left({{\varvec{\mu}}}_{{\varvec{i}}}\left({\varvec{t}}\right),{{\varvec{\Sigma}}}_{\mathbf{i}}\left({\varvec{t}}\right)\right).$$

Since the feature embeddings ${\varvec{V}}$ are engineered to approximately follow a multivariate normal distribution as described in the Producing feature embeddings section of the Supplementary Materials, it is reasonable to assume ${{\varvec{X}}}_{{\varvec{i}},{\varvec{t}}}$ to be a Gaussian process over time $t$. We further specify the mean and covariance functions ${{\varvec{\mu}}}_{{\varvec{i}}}({\varvec{t}})$ and ${{\varvec{\Sigma}}}_{\mathbf{i}}\left({\varvec{t}}\right)$ respectively. For some parameters ${{\varvec{\theta}}}_{\mathrm{GP}}=\{{{\varvec{\mu}}}_{0}, {{\varvec{\mu}}}_{1}, {{\varvec{\mu}}}_{2}, {{\varvec{\mu}}}_{3},{{\varvec{\mu}}}_{4}, {{\varvec{\mu}}}_{5},{{\varvec{\mu}}}_{{\varvec{H}}}, {{\varvec{\mu}}}_{{\varvec{Y}}{\varvec{H}}}, {\sigma }_{k},{\alpha }_{k},{\tau }_{k},{\rho }_{kl}, k=1,\dots ,p;l=1,\dots ,p\}$, we assume:

$${{\varvec{\mu}}}_{{\varvec{i}}}\left({\varvec{t}}\right)=E\left({{\varvec{X}}}_{{\varvec{i}},{\varvec{t}}}\right)={{\varvec{\mu}}}_{0}\left(1-{Y}_{i,t}\right)+{{\varvec{\mu}}}_{1}{Y}_{i,t}+{{\varvec{\mu}}}_{{\varvec{H}}}{H}_{i}+{{\varvec{\mu}}}_{{\varvec{Y}}{\varvec{H}}}{H}_{i}{Y}_{i,t}+{{\varvec{\mu}}}_{2}t+{{\varvec{\mu}}}_{3}\mathrm{log}t+{{\varvec{\mu}}}_{4}{Y}_{i,t}t+{{\varvec{\mu}}}_{5}{Y}_{i,t}\mathrm{log}t,$$

$$\mathrm{Var}\left({X}_{i,t,k}\right)={\sigma }_{k}^{2}\mathrm{exp}\left(2{\alpha }_{k}{H}_{i}\right), \mathrm{Cov}\left({X}_{i,t,k},{X}_{i,t,l}\right)={{\rho }_{kl}\sigma }_{k}{\sigma }_{l}\mathrm{exp}\{\left({\alpha }_{k}{+{\alpha }_{l})H}_{i}\right\}.$$

In summary, we assume that patient $i$’s expected embedding at time $t$, ${{\varvec{\mu}}}_{{\varvec{i}}}({\varvec{t}})$, is a function of ${Y}_{it}$, ${H}_{i}$, and $t$. We assume that the marginal variance of embedding component $k$ can be represented by some baseline ${\sigma }_{k}^{2}$ scaled by ${H}_{i}$. We denote the correlation between embedding components $k$ and $l$ as ${\rho }_{kl}$, which we assume to be constant over time. Between timepoints, we employ a first-order univariate autoregressive (AR(1)) kernel structure such that the residual at $t$, ${\epsilon }_{i,t,k}={X}_{i,t,k}-E({X}_{i,t,k}|{{\varvec{Y}}}_{{\varvec{i}}},{H}_{i})$, is a linear function of its preceding value ${\epsilon }_{i,t-1,k}$ with autocorrelation coefficient ${\tau }_{k}$:

$$E\left[{\epsilon }_{i,t,k}|{\epsilon }_{i,t-1,k}\right]=r{\tau }_{k}{\epsilon }_{i,t-1,k}.$$

$r\in [\mathrm{0,1}]$ is an autoregression regularization hyperparameter separately tuned via fivefold cross-validation maximizing the AUROC of ${Y}_{i,t}$ predictions: $r=0$ ignores intertemporal correlation while $r=1$ denotes undampened autoregression. We chose first-degree autoregression over higher-degree models due to computational ease and mitigation of overfitting. We provide a sensitivity analysis with respect to the choice of *k-*fold cross-validation in Supplementary Fig. S2 that demonstrates no significant effect of *k* on predictive accuracy.

#### Implementation and inference

MGP is fit via one iteration of an approximating expectation–maximization (EM) algorithm. We approximate the expected log-likelihood in the E-step using the marginal posterior of each latent phenotype state, ${\widehat{Y}}_{i,t}|{{\varvec{X}}}_{i}\boldsymbol{ }\forall t\in \{1,\dots ,{T}_{i}\}$, rather than the more complex joint posterior, ${\widehat{{\varvec{Y}}}}_{i}|{{\varvec{X}}}_{i}$. Before we fit MGP, we optimize $r$ using fivefold cross-validation on the labeled set as described above. We then initialize the EM by tuning the model parameters $\{{{\varvec{\lambda}}}_{\mathrm{markov}}, {{\varvec{\theta}}}_{\mathrm{GP}}\}$: ${{\varvec{\lambda}}}_{\mathrm{markov}}$ using simple logistic regression and ${{\varvec{\theta}}}_{\mathrm{GP}}$ using generalized least squares. Specific details of these tuning procedures are supplied in the Fitting MGP using Expectation–Maximization (EM) section of the Supplementary Materials. We then use this trained model to impute labels for the unlabeled set. We refer to predictions using this initial model as MGP’s supervised estimator ${\widehat{{\varvec{p}}}}_{{\varvec{s}}{\varvec{u}}{\varvec{p}}}$. Finally, we re-optimize $\{{{\varvec{\lambda}}}_{\mathrm{markov}}, {{\varvec{\theta}}}_{\mathrm{GP}}\}$ using both observed and imputed labels. We refer to predictions using this re-trained model as MGP’s semi-supervised estimator ${\widehat{{\varvec{p}}}}_{{\varvec{s}}{\varvec{e}}{\varvec{m}}{\varvec{i}}{\varvec{s}}{\varvec{u}}{\varvec{p}}}$. We execute one iteration of this EM procedure rather than running it to convergence for several reasons. First, since the EM is initialized at the consistent supervised estimator, the solution obtained after one iteration is guaranteed to be consistent assuming correct model specification while heuristically minimizing the influence of the unlabeled set. The one-step update also greatly reduces the computational cost. As Supplementary Fig. S3 demonstrates, SAMGEP’s performance is not sensitive to the maximum number of EM iterations allowed; in fact, increasing the number of iterations may marginally degrade performance when there exists sampling bias between the labeled and unlabeled sets, as is the case for our MS dataset.

### Combining semi-supervised and supervised predictions

Semi-supervised generative models such as MGP should benefit from the additional information in the unlabeled set if the model is correctly specified. However, semi-supervised predictors have been shown to be more sensitive to model misspecification than their supervised counterparts. To mitigate this effect, SAMGEP returns a weighted average of

3 $${\widehat{p}}_{sup}\,\,\mathrm{ and }\,\,{\widehat{p}}_{semisup}, {\widehat{p}}_{SAMGEP}=\alpha {\widehat{p}}_{semisup}+\left(1-\alpha \right){\widehat{p}}_{sup,}$$

with weight $\alpha$ selected by fivefold cross-validation maximizing the AUROC of ${Y}_{i,t}$ predictions. As Supplementary Fig. S1 shows, ${\widehat{{\varvec{p}}}}_{{\varvec{S}}{\varvec{A}}{\varvec{M}}{\varvec{G}}{\varvec{E}}{\varvec{P}}}$ never underperforms either ${\widehat{{\varvec{p}}}}_{{\varvec{s}}{\varvec{u}}{\varvec{p}}}$ or ${\widehat{{\varvec{p}}}}_{{\varvec{s}}{\varvec{e}}{\varvec{m}}{\varvec{i}}{\varvec{s}}{\varvec{u}}{\varvec{p}}}$, demonstrating SAMGEP’s insensitivity to sampling bias between the labeled and unlabeled sets.

### Data and metrics for evaluation

#### Simulation study

We generated datasets of $p=150$ count features along with $H$ for $N\in \{\mathrm{1000,5000,20000}\}$ unlabeled patients, each with a mean of $E\left[{T}_{i}\right]=25$ timepoints. To assess SAMGEP’s robustness to various model misspecifications, we varied the following generative parameters: (i) $Y|T$ where ‘independent’ indicates $Y\perp T$, ‘correct’ follows SAMGEP’s generative model, and ‘complex’ denotes over-parametrization of $Y(T)$; and (ii) ${\varvec{C}}|Y$ (marginally lognormal vs. log-t with 5 degrees of freedom). We considered $n=100$ labeled patients and let the number of informative features vary from 5 to 100. Details of our simulation generative mechanisms are supplied in the *Simulation Data Generative Mechanisms* section of the Supplementary Materials and Supplementary Table S1.

#### Multiple sclerosis (MS) relapse and heart failure (HF) onset identification

We further validate SAMGEP’s performance by classifying MS relapse and HF status over time using two EHR studies. Whereas HF is a chronic disease for which primary interest lies in the cumulative probability of disease over time, MS is a relapsing and remitting phenotype for which we seek to predict all relapses over time. For identification of MS relapse, we collected EHR data between January 1, 2006 and December 31, 2016 for 4706 patients with at least one MS ICD code from the Research Patient Data Registry (RPDR) of the Mass General Brigham (MGB) health system in Boston, MA. We derived neurologist-confirmed MS relapse events and dates for 1435 patients from the Comprehensive Longitudinal Investigation of Multiple Sclerosis (CLIMB) research registry. For this study, relapse was defined as a clinical and/or radiological event. Clinical relapse was defined as having new or recurrent MS-related neurological symptoms lasting persistently for at least 24 h without fever or infection. Radiological relapse was defined as having either a new T1-enhancing lesion and/or a new or enlarging T2-FLAIR hyperintense lesion on the brain, orbit, or spinal cord MRI according to the clinical radiology report. 57.2% of patients in CLIMB had at least one relapse event, with a mean of 2.60 relapses per patient and 0.081 relapses per patient-month. For HF, we collected EHR data for 59,395 patients in the MGB RPDR with at least one ICD code for HF. We compiled HF status and onset dates for 300 randomly selected patients from this cohort via chart review by two independent cardiologists at MGB who jointly reconciled any differences in initial assessment. Incident HF was defined using a previously validated algorithm that includes at least 1 HF ICD-9 code (425.x or 428.x) combined with use of an intravenous diuretic within 90 days of the above HF code^34^.

Among the 300, 60.7% developed HF during follow-up. The MGB IRB approved the use of both EHR and research registry data, and data were appropriately deidentified before use in accordance with relevant guidelines and regulations. Informed consent was obtained from all subjects and/or their legal guardians by RPDR investigators during collection of the data.

From the EHR dataset we extracted age, sex, and patient-level occurrences of ICD-9 diagnosis codes, RxNorm prescription codes, and CPT procedures codes. We mapped ICD-9 codes to PheCodes using the established PheWAS mapping in order to better represent clinical conditions and improve generalizability^35^. In addition to PheCodes, RxNorm codes, and CPT codes, our feature set included clinical concepts mapped to concept unique identifiers (CUIs) extracted from free-text clinical narratives via the Narrative Information Linear Extraction (NILE) natural language processing (NLP) method^36^. We binned all EHR features into consecutive, non-overlapping 1-month time intervals such that ${{\varvec{C}}}_{{\varvec{i}},{\varvec{t}}}$ represents the counts of patient $i$’s PheCodes, CPT codes, and NLP features between months $t$ and $t+1$. As Supplementary Figure S4 demonstrates, SAMGEP attains higher accuracy with longer window lengths, suggesting that the user should generally employ the longest window length that achieves sufficient temporal precision for a given task. For identification of MS relapse, we selected 154 features via marginal screening of association between all candidate features and 1-year relapse risk as described in Ahuja et al.^37^ For HF onset identification, we selected 121 features with embedding cosine similarities of 0.1 or above relative to the HF ICD code. Selected features are displayed in the Supplementary Materials.

#### Benchmark methods for comparison

We chose benchmark methods based on a comprehensive literature review of supervised and/or semisupervised temporal process prediction methods using longitudinal EHR data. We considered as benchmarks three supervised methods using the labeled set alone: (i) long short term memory RNN (LSTM)^24,39,43,44^ trained with ${{\varvec{C}}}_{{\varvec{i}},{\varvec{t}}}$, (ii) RETAIN^26^ trained with raw EHR observations in the continuous time domain, and (iii) linear discriminant analysis (LDA) trained with patient-timepoint embeddings generated without weights (${{\varvec{X}}}_{{\varvec{i}},{\varvec{t}}}^{0}={{\varvec{C}}}_{{\varvec{i}},{\varvec{t}}}{\varvec{V}}$), which we refer to as LDA_embed_. LDA_embed_ predicts ${Y}_{i,t}$ using only concurrent features without considering the time sequence. In addition, we considered a semi-supervised benchmark: HMM^26–29,45,46^ with a multivariate gaussian emission trained on ${{\varvec{X}}}_{{\varvec{i}},{\varvec{t}}}^{0}$. As a baseline we also included predictions based only on the closest PheCodes (MS: 355; HF: 428). See the Benchmark method implementation details section of the Supplementary Materials for details of our benchmark method implementations. In Supplementary Fig. S5 we also include results for LASSO-penalized logistic regression^16,17,34,37–39^, random forest (RF)^40,41^, and LDA^47^ trained with ${{\varvec{C}}}_{{\varvec{i}},{\varvec{t}}}$. These methods leverage neither the time sequence nor ${\varvec{V}}$ and achieve subpar predictive accuracy.

#### Evaluation metrics

We computed various performance metrics for SAMGEP and benchmark methods using a held-out validation set ${\{{\varvec{C}}}_{{\varvec{v}}{\varvec{a}}{\varvec{l}}},{{\varvec{Y}}}_{val}\}$. To evaluate methods’ predictions for ${Y}_{i,t}$, we computed (i) AUC and (ii) F1 score choosing a cutoff that achieves 95% specificity. Let ${N}_{i}\left(t\right)={\sum }_{k\le t}{Y}_{i,k}(1-{Y}_{i,k-1})$ denote the observed all-event counting process, where ${Y}_{i,0}=0$, and let ${F}_{i}\left(t\right)=1-{\Pi }_{k\le t}(1-{Y}_{i,k})$ denote the observed first-event process. We evaluated methods’ phenotype counting process predictions by computing the area between ${N}_{i}\left(t\right)$ and the predicted counting process ${\widehat{N}}_{i}\left(t\right)={\sum }_{k\le t}{I(\widehat{\pi }}_{i,k}\ge c)$, denoted as ABC_count_, where ${\widehat{\pi }}_{i,k}$ denotes a method’s prediction of $P({Y}_{i,k}=1)$ and $c$ is chosen such that in labeled set

$$\frac{{\sum }_{i=1}^{n}{\sum }_{k=1}^{T(i)}{I(\widehat{\pi }}_{i,k}\ge c)}{{\sum }_{i=1}^{n}T(i)}=\frac{{\sum }_{i=1}^{n}{\sum }_{k=1}^{T(i)}{Y}_{i,k}}{{\sum }_{i=1}^{n}T(i)}.$$

Likewise, we evaluated methods’ first-event cumulative probability (CDF) predictions by computing the area between ${F}_{i}\left(t\right)$ and the predicted CDF ${\widehat{F}}_{i,t}=1-{\prod }_{k\le t}(1-{\widehat{\lambda }}_{i,k})$, denoted by ABC_cdf_, where ${\lambda }_{i,k}$ denotes patient $i$’s true hazard at time $k$ and ${\widehat{\lambda }}_{i,k}$ denotes a method’s prediction thereof. In the absence of censoring, ABC_cdf_ is equivalent to the mean absolute difference between true and predicted event times. Since SAMGEP and HMM jointly model the outcome sequence $\{{Y}_{i,1},\dots ,{Y}_{i,T\left(i\right)}\}$, we used these methods to directly estimate ${\widehat{F}}_{i,t}$. Other methods only predict marginal probabilities ${\widehat{\pi }}_{i,t},$ so we assumed that ${{\widehat{\lambda }}_{i,t}=\widehat{\pi }}_{i,t}$, or equivalently that event states are independent over time. Rather than report raw ABC quantities—whose scale can vary greatly across settings—we report methods’ percent decrease below those of the null model that sets ${\widehat{\pi }}_{ik}$ to the prevalence at time *k*:

4 $${\mathrm{ABC}}_{\mathrm{cdf}}^{\mathrm{Gain}}= \frac{({\mathrm{ABC}}_{\mathrm{cdf},\mathrm{null}}-{\mathrm{ABC}}_{\mathrm{cdf},\mathrm{method}})}{{\mathrm{ABC}}_{\mathrm{cdf},\mathrm{null}}}, \mathrm{and} \,\,{\mathrm{ABC}}_{\mathrm{count}}^{\mathrm{Gain}}= \frac{({\mathrm{ABC}}_{\mathrm{count},\mathrm{null}}-{\mathrm{ABC}}_{\mathrm{count},\mathrm{method}})}{{\mathrm{ABC}}_{\mathrm{count},\mathrm{null}}}.$$

We do not compute ${\mathrm{ABC}}_{\mathrm{count}}^{\mathrm{Gain}}$ for HF onset identification since only the CDF is of interest for HF. Finally, for both MS relapse and HF onset identification, we qualitatively evaluate SAMGEP’s feature selection and weighting mechanism by generating feature clouds using the product of SAMGEP’s feature weights and emprirical feature standard devations: ${w}_{j}={W}_{j,j}\times \widehat{s}({{\varvec{C}}}_{,j})$.

## Supplementary Information

Supplementary Information.

## Data Availability

The Electronic Health Record (EHR) and research registry data underlying this article were provided by Mass General Brigham (MGB) Research Information Sciences & Computing by permission, and with the approval of the MGB Institutional Review Board (IRB). These data will be shared on request to the corresponding author with permission of MGB Research Information Sciences & Computing as well as the MGB IRB.

## References

[1] Kohane IS, Churchill SE, Murphy SN (2012). A translational engine at the national scale: Informatics for integrating biology and the bedside. J. Am. Med. Inform. Assoc..

[2] Hripcsak G, Albers DJ (2012). Next-generation phenotyping of electronic health records. J. Am. Med. Inform. Assoc..

[3] Miotto R, Li L, Kidd BA, Dudley JT (2016). Deep patient: An unsupervised representation to predict the future of patients from the electronic health records. Sci. Rep..

[4] Liao KP (2010). Electronic medical records for discovery research in rheumatoid arthritis. Arthritis Care Res..

[5] Cipparone CW (2015). Inaccuracy of ICD-9 codes for chronic kidney disease: A study from two practice-based research networks (PBRNs). J. Am. Board Fam. Med..

[6] Uno H (2018). Determining the time of cancer recurrence using claims or electronic medical record data. JCO Clin. Cancer Inform..

[7] Hassett MJ (2017). Detecting lung and colorectal cancer recurrence using structured clinical/administrative data to enable outcomes research and population health management. Med. Care.

[8] Chubak J (2012). Administrative data algorithms to identify second breast cancer events following early-stage invasive breast cancer. J. Natl. Cancer Inst..

[9] Carroll RJ (2012). Portability of an algorithm to identify rheumatoid arthritis in electronic health records. J. Am. Med. Inform. Assoc..

[10] Liao KP (2015). Methods to develop an electronic medical record phenotype algorithm to compare the risk of coronary artery disease across 3 chronic disease cohorts. PLoS ONE.

[11] Liao K (2019). High-throughput multimodal automated phenotyping (MAP) with application to PheWAS. J. Am. Med. Inform. Assoc..

[12] Ahuja Y (2020). sureLDA: A multidisease automated phenotyping method for the electronic health record. J. Am. Med. Inform. Assoc..

[13] Beaulieu-Jones BK, Greene CS (2016). Semi-supervised learning of the electronic health record for phenotype stratification. J. Biomed. Inform..

[14] Newton KM (2013). Validation of electronic medical record-based phenotyping algorithms: Results and lessons learned from the eMERGE network. J. Am. Med. Inform. Assoc..

[15] Ananthakrishnan AN (2013). Improving case definition of Crohn’s disease and ulcerative colitis in electronic medical records using natural language processing: a novel informatics approach. Inflamm. Bowel Dis..

[16] Xia Z (2013). Modeling disease severity in multiple sclerosis using electronic health records. PLoS ONE.

[17] Liao KP (2015). Development of phenotype algorithms using electronic medical records and incorporating natural language processing. BMJ.

[18] Kirby JC (2016). PheKB: A catalog and workflow for creating electronic phenotype algorithms for transportability. J. Am. Med. Inform. Assoc..

[19] Halpern, Y., Choi, Y., Horng, S. & Sontag, D. Using anchors to estimate clinical state without labeled data. In *AMIA Annual Symposium Proceedings* vol. 2014 606 (2014). PMC4419996 25954366

[20] Yu S (2017). Enabling phenotypic big data with PheNorm. J. Am. Med. Inform. Assoc..

[21] Choi, E., Du, N., Chen, R., Song, L. & Sun, J. Constructing disease network and temporal progression model via context-sensitive hawkes process. In *Proc.-IEEE Int. Conf. Data Mining, ICDM* **2016**-**Janua**, 721–726 (2016).

[22] Kaji DA (2019). An attention based deep learning model of clinical events in the intensive care unit. PLoS ONE.

[23] Rajkomar A (2018). Scalable and accurate deep learning with electronic health records. NPJ Digit. Med..

[24] Ruan T (2019). Representation learning for clinical time series prediction tasks in electronic health records. BMC Med. Inform. Decis. Mak..

[25] Cheng, Y., Wang, F., Zhang, P. & Hu, J. Risk prediction with electronic health records: A deep learning approach. In *16th SIAM Int. Conf. Data Min. 2016, SDM 2016* 432–440 (2016) 10.1137/1.9781611974348.49.

[26] Choi E (2016). RETAIN: An interpretable predictive model for healthcare using reverse time attention mechanism. Adv. Neural Inf. Process. Syst..

[27] Pivovarov R (2015). Learning probabilistic phenotypes from heterogeneous EHR data. J. Biomed. Inform..

[28] Pivovarov, R. *Electronic Health Record Summarization Over Heterogeneous and Irregularly Sampled Clinical Data* (Columbia University, 2016).

[29] Jackson CH, Sharples LD, Thompson SG, Duffy SW, Couto E (2003). Multistate Markov models for disease progression with classification error. Stat..

[30] Sukkar, R., Katz, E., Zhang, Y., Raunig, D. & Wyman, B. T. Disease progression modeling using Hidden Markov Models. In *Conf Proc IEEE Eng Med Biol Soc* 2845–2848 (2012). 10.1109/EMBC.2012.6346556 23366517

[31] Wang, X., Sontag, D. & Wang, F. Unsupervised learning of disease progression models. In *Proc. ACM SIGKDD Int. Conf. Knowl. Discov. Data Min.* 85–94 (2014). 10.1145/2623330.2623754.

[32] Zhou X, Kang K, Song X (2020). Two-part hidden Markov models for semicontinuous longitudinal data with nonignorable missing covariates. Stat. Med..

[33] Yu S (2017). Surrogate-assisted feature extraction for high-throughput phenotyping. J. Am. Med. Inform. Assoc..

[34] Barnardo A, Casey C, Carroll RJ, Wheless L, Denny JCCL (2017). Developing electronic health record algorithms that accurately identify patients with systemic lupus erythematosus. Arthritis Care Res..

[35] Denny JC (2013). Systematic comparison of phenome-wide association study of electronic medical record data and genome-wide association study data. Nat. Biotechnol..

[36] Yu, S., Cai, T. & Cai, T. NILE: Fast natural language processing for electronic health records. *arXiv* 1–23 (2013).

[37] Cai T (2018). Association of interleukin 6 receptor variant with cardiovascular disease effects of interleukin 6 receptor blocking therapy: A phenome—Wide association study. JAMA Cardiol..

[38] Lin C (2013). Automatic prediction of rheumatoid arthritis disease activity from the electronic medical records. PLoS ONE.

[39] Li R (2019). Detection of bleeding events in electronic health record notes using convolutional neural network models enhanced with recurrent neural network autoencoders: Deep learning approach. J. Med. Internet Res..

[40] Yang Z, Dehmer M, Yli-Harja O, Emmert-Streib F (2020). Combining deep learning with token selection for patient phenotyping from electronic health records. Sci. Rep..

[41] Sun Z (2019). A probabilistic disease progression modeling approach and its application to integrated Huntington’s disease observational data. JAMA Open.

[42] Verma, A., Powell, G., Luo, Y., Stephens, D. & Buckeridge, D. L. Modeling disease progression in longitudinal EHR data using continuous-time hidden Markov models. 1–5 (2018). 10.3233/SHTI190358 31438058

[43] Castro VM (2015). Validation of electronic health record phenotyping of bipolar disorder and controls. Am. J. Psychiatry.

[44] Anderson AE (2016). Electronic health record phenotyping improves detection and screening of type 2 diabetes in the general United States population: A cross-sectional, unselected, retrospective study. J. Biomed. Inform..

[45] Garg R, Dong S, Shah S, Jonnalagadda SR (2016). A Bootstrap Machine Learning Approach to Identify Rare Disease Patients from Electronic Health Records Division of Health and Biomedical Informatics.

[46] Teixeira PL (2017). Evaluating electronic health record data sources and algorithmic approaches to identify hypertensive individuals. J. Am. Med. Inform. Assoc..

[47] Yang S (2018). Early detection of disease using electronic health records and fisher’s wishart discriminant analysis. Proc. Comput. Sci..

