# Supplementary material for: A semi-supervised adaptive Markov Gaussian embedding process (SAMGEP) for prediction of phenotype event times using the electronic health record
Source: Sci Rep. 2022 Oct 22;12:17737. doi: 10.1038/s41598-022-22585-3 (PMC9588081; doi:10.1038/s41598-022-22585-3)
Supplement: Supplementary file 1 — Supplementary Information. [file 41598_2022_22585_MOESM1_ESM.zip › SAMGEP Supplementary Materials/SAMGEP_supplement_final_scientific_reports_R1.pdf]

SUPPLEMENTARY MATERIALS

Producing Feature Embeddings

Feature-level embedding vectors were produced by applying singular value decomposition (SVD) to the pointwise mutual information (PMI) matrix of feature occurrence/co-occurrence counts in the Partners HealthCare EHR database. Co-occurrence was defined as the presence of two features in a patient's chart within 1 week of each other. Denoting the marginal occurrence rates of features i and j as p_i and p_j respectively, and the co-occurrence rate of features i and j as p_{ij}, the PMI of the two features is defined as

$$pmi(i,j) = \log\left(\frac{p_{ij}}{p_i p_j}\right)$$

Sensibly, PMI is negative if features i and j are negatively associated, positive if positively associated, and zero if independent. Decomposing the PMI matrix rather than the co-occurrence count matrix is beneficial as PMI normalizes feature prevalence, preventing outsized influence of common features such as the word 'patient' in clinical notes. Since the PMI matrix is symmetric, SVD is simply an eigendecomposition thereof, and the initial embedding vector $\boldsymbol{V_{j,0}}$ for feature j is simply feature j's co-occurrence vector projected onto each PMI eigenvector. For this analysis we took only the first 1,000 eigenvectors, yielding 1,000-dimensional initial embeddings. To further reduce the embedding dimension, we performed PCA on the set of initial embedding vectors corresponding to our assembled features and took the first 10 PC scores for feature j as its final embedding vector $\boldsymbol{V_j}$.

Fitting MGP using Expectation-Maximization (EM)

Initialization: All parameters are initialized on the labeled set. MGP's log-likelihood function can be decomposed into three components: $\log P(\boldsymbol{Y_1})$, $\log P(\boldsymbol{Y_t}|\boldsymbol{Y_{t-1}})$, and $\log f(\boldsymbol{X}|\boldsymbol{Y})$. The first component can be expressed as:

$$\log P(\boldsymbol{Y_1}) = \sum_{i=1}^{N} Y_{i,1} \log \pi_{init} + (1 - Y_{i,t})(1 - \log \pi_{init})$$

where $\pi_{init} = expit(\lambda_{init} + \lambda_{H0} H_i^{log})$. We recognize this is as the objective function of a logistic regression with outcome $\boldsymbol{Y_1}$ and predictor $\boldsymbol{H^{log}}$, and thus fit $\{\lambda_{init}, \lambda_{H0}\}$ using the *glm* package in *R* with binomial outcome and logit link function. Likewise, the second component can be expressed as:

$$\log P(\boldsymbol{Y_t}|\boldsymbol{Y_{t-1}}) = \sum_{i=1}^{N} \sum_{t=2}^{T(i)} Y_{i,t} \log \pi_t + (1 - Y_{i,t}) \log(1 - \pi_t)$$

where $\pi_t = expit(\lambda_0(1 - Y_{t-1}) + \lambda_1 Y_{t-1} + \lambda_H H_i^{log} + \lambda_t t + \lambda_{logt} \log t)$. This is the objective function of a logistic regression predicting $\boldsymbol{Y_{2:T(i)}}$ from previous phenotype states, $\boldsymbol{H^{log}}$, and t, so we fit $\{\lambda_0, \lambda_1, \lambda_H, \lambda_t, \lambda_{logt}\}$ once again using R's *glm* package. Finally, the third component is by design the log-likelihood of a generalized least squares model with outcome $\boldsymbol{X}$ and mean/covariance specified in the "Gaussian Process Assumption" subsection of the Methods section. We fit the mean model and marginal variance parameters $\{\boldsymbol{\mu_0}, \boldsymbol{\mu_1}, \boldsymbol{\mu_H}, \boldsymbol{\mu_{YH}}, \boldsymbol{\mu_t}, \boldsymbol{\mu_{Yt}}, \sigma_{1:m}, \alpha_{1:m}\}$ using the *gls* package in R with first-order autoregression. We compute the maximum likelihood estimator of the intra-temporal correlation parameters $\boldsymbol{\rho} \in R^{m \times m}$ using the $H_i^{\alpha_k}$-normalized residuals of the *gls* fit, $\boldsymbol{\hat{\epsilon}_{i,t}} \in \boldsymbol{R^m}$, as follows:

$$\hat{\rho}_{kl} = \frac{1}{NT\sigma_k \sigma_l} \sum_{i=1}^{N} \sum_{t=1}^{T(i)} \hat{\epsilon}_{i,t,k} \hat{\epsilon}_{i,t,l}$$

Finally, we estimate the inter-temporal autocorrelation parameters $\boldsymbol{\tau} \in R^m$ using component-wise ordinary least squares (OLS) regression of $\boldsymbol{\hat{\epsilon}_{i,t,k}}$ versus $\boldsymbol{\hat{\epsilon}_{i,t-1,k}}$ $\forall\, k \in \{1, \ldots, m\}$. Note that unlike standard vector autoregression, here we assume that $\hat{\epsilon}_{i,t,k} | \hat{\epsilon}_{i,t',k} \perp \hat{\epsilon}_{i,t',l}\ \forall\, t' \neq t, k \neq l$.

E-step: Let $\hat{p}_{it} = E[Y_{i,t}|\boldsymbol{X}]$. Again, we estimate the marginal posterior $Y_{i,t}|\boldsymbol{X}$ rather than the joint $\boldsymbol{Y_{i,1:T(i)}}|\boldsymbol{X}$, which dramatically improves computational efficiency at the expense of being unable to re-optimize intertemporal parameters. Since both Markov Process and first-order autoregression assume that

a timepoint is independent of the past and future conditional on its neighboring timepoints, we can accurately approximate $\hat{p}_{i,t}$ as $E\left[Y_{i,t}\middle|\boldsymbol{X}_{\boldsymbol{i},\boldsymbol{t}-\boldsymbol{1}},\boldsymbol{X}_{\boldsymbol{i},\boldsymbol{t}},\boldsymbol{X}_{\boldsymbol{i},\boldsymbol{t}+\boldsymbol{1}}\right]$ rather than $E\left[Y_{i,t}\middle|\boldsymbol{X}_{\boldsymbol{i},\boldsymbol{1}:\boldsymbol{T}(\boldsymbol{i})}\right]$:

$$\hat{p}_{i,t} = \frac{\sum_{u=0}^{1}\sum_{w=0}^{1} P\left(Y_{i,t-1}=u\right)P\left(Y_{i,t}=1\middle|Y_{i,t-1}=u\right)P\left(Y_{i,t+1}=w\middle|Y_{i,t}=1\right)f\left(\boldsymbol{X}_{\boldsymbol{i},\boldsymbol{t}-\boldsymbol{1}},\boldsymbol{X}_{\boldsymbol{i},\boldsymbol{t}},\boldsymbol{X}_{\boldsymbol{i},\boldsymbol{t}+\boldsymbol{1}}\middle|Y_{i,t-1},Y_{i,t},Y_{i,t+1}\right)}{\sum_{u=0}^{1}\sum_{v=0}^{1}\sum_{w=0}^{1} P\left(Y_{i,t-1}=u\right)P\left(Y_{i,t}=v\middle|Y_{i,t-1}=u\right)P\left(Y_{i,t+1}=w\middle|Y_{i,t}=v\right)f\left(\boldsymbol{X}_{\boldsymbol{i},\boldsymbol{t}-\boldsymbol{1}},\boldsymbol{X}_{\boldsymbol{i},\boldsymbol{t}},\boldsymbol{X}_{\boldsymbol{i},\boldsymbol{t}+\boldsymbol{1}}\middle|Y_{i,t-1},Y_{i,t},Y_{i,t+1}\right)}$$

Note that $P\left(Y_{i,t-1}=u\right)$ here is a marginal probability, independent of $\{\boldsymbol{X}_{\boldsymbol{i}}, Y_i\}_{1:t-2}$. While this is misspecified, it is faster and indeed achieves higher test set accuracy in our real-world EHR example than jointly estimating $E\left[\boldsymbol{Y}_{\boldsymbol{i},\boldsymbol{1}:\boldsymbol{T}(\boldsymbol{i})}\middle|\boldsymbol{X}_{\boldsymbol{i},\boldsymbol{1}:\boldsymbol{T}(\boldsymbol{i})}\right]$. $f\left(\boldsymbol{X}_{i,t-1},\boldsymbol{X}_{i,t},\boldsymbol{X}_{i,t+1}\middle|Y_{i,t-1},Y_{i,t},Y_{i,t+1}\right)$ is simply the density of the multivariate normal specified in the "Gaussian Process Assumption" subsection. Finally, for the endpoints $t=\{1,T(i)\}$, we respectively omit $\left\{\boldsymbol{X}_{\boldsymbol{i},\boldsymbol{t}-\boldsymbol{1}},Y_{i,t-1}\right\}$ and $\left\{\boldsymbol{X}_{\boldsymbol{i},\boldsymbol{t}+\boldsymbol{1}},Y_{i,t+1}\right\}$ in the above computation and predict $\hat{p}_{i,t}$ as above using the remaining two timepoints.

M-step: Re-optimizing the model parameters follows a similar procedure to initialization. The expected log-likelihood function can be decomposed into three components: $E[\log P(\boldsymbol{Y}_{\boldsymbol{1}})]$, $E[\log P(\boldsymbol{Y}_{\boldsymbol{t}}|\boldsymbol{Y}_{\boldsymbol{t}-\boldsymbol{1}})]$, and $E[\log f(\boldsymbol{X}|\boldsymbol{Y})]$. Note that since we only estimate marginal rather than joint posteriors in the E-step, we cannot derive a closed-form expression for the second component and therefore maintain the transition parameters $\{\lambda_0,\lambda_1,\lambda_H,\lambda_t\}$ at their initial values. Likewise, we cannot re-infer the autocorrelation parameters $\tau_{1:m}$ and thus maintain them at their initial values as well. The first component can be expressed as:

$$E[\log P(\boldsymbol{Y}_{\boldsymbol{1}})] = \sum_{i=1}^{N} \hat{p}_{i1}\log\pi_{init} + (1-\hat{p}_{i1})(1-\log\pi_{init})$$

We recognize this as the objective function of a weighted logistic regression with outcome $[\boldsymbol{0}_{\boldsymbol{N}},\boldsymbol{1}_{\boldsymbol{N}}]$, predictor $[\boldsymbol{H}^{\boldsymbol{log}},\boldsymbol{H}^{\boldsymbol{log}}]$, and observation weights $[\boldsymbol{1}-\widehat{\boldsymbol{p}}_{\boldsymbol{1}},\ \widehat{\boldsymbol{p}}_{\boldsymbol{1}}]$, and thus refit $\{\lambda_{init},\lambda_{H0}\}$ accordingly using the *glm* package. Similarly, the third component becomes the log-likelihood of a generalized least squares model with outcome $[\boldsymbol{X},\boldsymbol{X}]$, mean $[\boldsymbol{\mu}|\boldsymbol{Y}=\boldsymbol{0},\ \boldsymbol{\mu}|\boldsymbol{Y}=\boldsymbol{1}]$, covariance specified in the "Gaussian Process Assumption" subsection of the Methods, and observation weights $[\boldsymbol{1}-\widehat{\boldsymbol{p}}_{\boldsymbol{1}},\ \widehat{\boldsymbol{p}}_{\boldsymbol{1}}]$. We can thus refit $\{\boldsymbol{\mu}_{\boldsymbol{0}},\boldsymbol{\mu}_{\boldsymbol{1}},\boldsymbol{\mu}_{\boldsymbol{H}},\boldsymbol{\mu}_{\boldsymbol{YH}},\boldsymbol{\mu}_{\boldsymbol{t}},\boldsymbol{\mu}_{\boldsymbol{Yt}},\sigma_{1:m},\alpha_{1:m}\}$ using the *gls* package in R with first-order autocorrelation. Finally,

we re-estimate $\boldsymbol{\rho}$ using the $H_i^{\alpha_k}$-normalized residuals of the weighted *gls* fit, $[\hat{\boldsymbol{\epsilon}}_{\boldsymbol{i,t,Y0}}, \hat{\boldsymbol{\epsilon}}_{\boldsymbol{i,t,Y1}}] \in \boldsymbol{R^{2m}}$, where $\hat{\boldsymbol{\epsilon}}_{\boldsymbol{i,t,Y0}}$ denotes the residuals for observations where $\boldsymbol{Y} = \boldsymbol{0}$ and $\hat{\boldsymbol{\epsilon}}_{\boldsymbol{i,t,Y1}}$ where $\boldsymbol{Y} = \boldsymbol{1}$:

$$\hat{\rho}_{kl} = \frac{1}{NT\sigma_k\sigma_l}\sum_{i=1}^{N}\sum_{t=1}^{T(i)}(1 - \hat{p}_{i,t})\hat{\epsilon}_{i,t,Y0,k}\hat{\epsilon}_{i,t,Y0,l} + \hat{p}_{i,t}\hat{\epsilon}_{i,t,Y1,k}\hat{\epsilon}_{i,t,Y1,l}$$

Benchmark Method Implementation Details

For our LSTM comparator, we utilized a many-to-one architecture consisting sequentially of a 1D convolutional layer, the core LSTM layers, and a time-distributed dense layer, each with dropout and L2-regularization. We extensively optimized this structure in development, experimenting with different architectures (i.e. network depth, breadths of dense layers), dropout layer hyperparameters, and L1/L2 regularization hyperparameters, to maximize predictive AUROC on the real-world MS prediction task using 100 labels. Moreover, we present LSTM trained using $\boldsymbol{C}_{\boldsymbol{i,t}}$ rather than $\boldsymbol{X}_{\boldsymbol{i,t}}^{\boldsymbol{0}}$ as the former achieved higher AUROCs and F1 scores on the MS prediction task using 100 labels. Our implementation of RETAIN utilized bidirectional GRUs with L2 regularization and dropout at each layer. We tuned the model using Adamax with a learning rate of 0.003 and batch size of 64 for a maximum of 100 epochs; we found that RETAIN's performance was not particularly sensitive to these settings. For the HMM we initialized state transition probabilities and emission parameters using the labeled set, and trained the model using both labeled and unlabeled sets via the Baum-Welch algorithm. We optimized the L1 regularization hyperparameter for LASSO, and the max tree depth and forest size hyperparameters for RF, using 5-fold cross-validation maximizing AUROC for a given prediction task.

SAMGEP vs. Supervised and Unsupervised MGP

As Figure S1 demonstrates, SAMGEP's mechanism for adaptively weighting MGP's supervised and semi-supervised predictors consistently achieved results statistically equivalent if not superior to the individual predictors. For prediction of MS relapse, the supervised predictor tended to achieve higher

AUCs and F1 scores and the semi-supervised one tended to perform better per ABC_{cdf} and ABC_{count}. For prediction of HF onset, the semi-supervised predictor tended to perform better across metrics. SAMGEP's adaptive weighted average of the two achieved robust results for both outcomes.

Tuning SAMGEP's Hyperparameters

For SAMGEP we pre-optimized the following hyperparameters during model development: 1) the *k* of all *k*-fold cross-validation steps within the procedure, 2) the maximum number of EM iterations allowed, and 3) the window length used to pre-process the raw data into discrete timepoints. As Supplementary Figures S2, S3, and S4 depict, we found that SAMGEP's predictive accuracy is not very sensitive to the first two settings. While it is sensitive to the third, this simply reflects the fact that a larger window length results in more datapoints/information per time interval – a tradeoff of temporal precision for accuracy.

Simulation Data Generative Mechanisms

In our simulation study we generated datasets via a four-step procedure: for each patient, generate (i) the total number of timepoints T_i and (ii) the initial phenotype state $Y_{i,0}$; for timepoints $2{:}T_i$ generate (iii) $Y_{i,t}|Y_{i,t-1}$; and (iv) generate longitudinal feature counts $\boldsymbol{C_i}|\boldsymbol{Y_i}$. We vary the following generative parameters:

(1) The mechanism of $Y|T$, where 'independent' indicates that $Y \perp T$ (i.e. $Y_{i,t} \sim Bern(\pi_0\{H_i\}) \; \forall \, i,t$), 'correct' follows SAMGEP's generative mode (i.e. $Y_{i,t} \sim Bern(\pi_{i,t}), \pi_{i,t} = expit\{\lambda_0(1-y_{t-1}) + \lambda_1 y_{t-1} + \lambda_2 t + \lambda_3 \log t + \lambda_H H_i\}$), and 'complex' denotes over-parametrization of $Y(T)$ (i.e. $Y_{i,t} \sim Bern(\pi_{i,t}), \pi_{i,t} = expit\{\lambda_0(1-Y_{t-1}) + \lambda_1 y_{t-1} + \lambda_2 t + \lambda_3 \log t + \lambda_H H_i + \lambda_{02}(1-Y_{t-1})t + \lambda_{12} y_{t-1} t + \lambda_{03}(1-Y_{t-1}) \log t + \lambda_{13} Y_{t-1} \log t + \lambda_{0H}(1-Y_{t-1})H_i + \lambda_{1H} Y_{t-1} H_i + \lambda_{2H} H_i t + \lambda_{3H} H_i \log t\}$), with generative parameters $\boldsymbol{\lambda}$ optimized using our real-world MS relapse dataset;

(2) The marginal distribution of $\boldsymbol{C_i}|\boldsymbol{Y_i}$, where 'lognormal' indicates that marginally $\log C_{i,j,t}\,|Y_{i,t} \sim N\left(\alpha_{0,j}\left(1 - Y_{i,t}\right) + \alpha_{1,j}Y_{i,t}, \sigma^2\right)$ and 'log-t' that $\log C_{i,j,t}\,|Y_{i,t} \sim t\left(\alpha_0\left(1 - Y_{i,t}\right) + \alpha_{1,j}Y_{i,t}, 5df\right)$;

(3) The inter-temporal correlation parameter ρ of $\boldsymbol{C}|\boldsymbol{Y}$, where $cor\left(C_{i,t}, C_{i,s}\middle|\boldsymbol{Y}\right) = \rho^{|t-s|}$

(4) The number of observed phenotype labels n;

(5) The total number of patients N;

(6) The number of informative features, where the generative β coefficients of any non-informative features are set to 0.

In summary, we generated datasets per the mechanisms outlined in the following table. Figure S5 demonstrates results for SAMGEP and all benchmarks from this Simulation Study.

$\boldsymbol{Y}\|\boldsymbol{T}$	$\boldsymbol{C}\|\boldsymbol{Y}$	$\boldsymbol{\rho}$	$\boldsymbol{n}$	$\boldsymbol{N}$	***n*Informative**
Correct	Lognormal	0.4	100	5000	20
Complex	Lognormal	0.4	100	5000	20
Independent	Lognormal	0.4	100	5000	20
Correct	Log-t	0.4	100	5000	20
Correct	Lognormal	0	100	5000	20
Correct	Lognormal	0.8	100	5000	20
Correct	Lognormal	0.4	50	5000	20
Correct	Lognormal	0.4	200	5000	20
Correct	Lognormal	0.4	100	1000	20
Correct	Lognormal	0.4	100	20000	20
Correct	Lognormal	0.4	100	5000	5
Correct	Lognormal	0.4	100	5000	100

Table S1: Generative mechanism parameters used in the Simulation Study

	**CLIMB (s.e.)**	**Non-CLIMB (s.e.)**	**p-value**
**Timepoint Prevalence**	0.149 (0.008)	0.119 (0.008)	0.010
**Patient Prevalence**	0.610 (0.056)	0.431 (0.048)	0.015

Table S2: Timepoint and patient prevalences among patients in the CLIMB cohort versus those not in CLIMB. P-values were computed using Wald tests.

SUPPLEMENTARY FIGURES

(A)

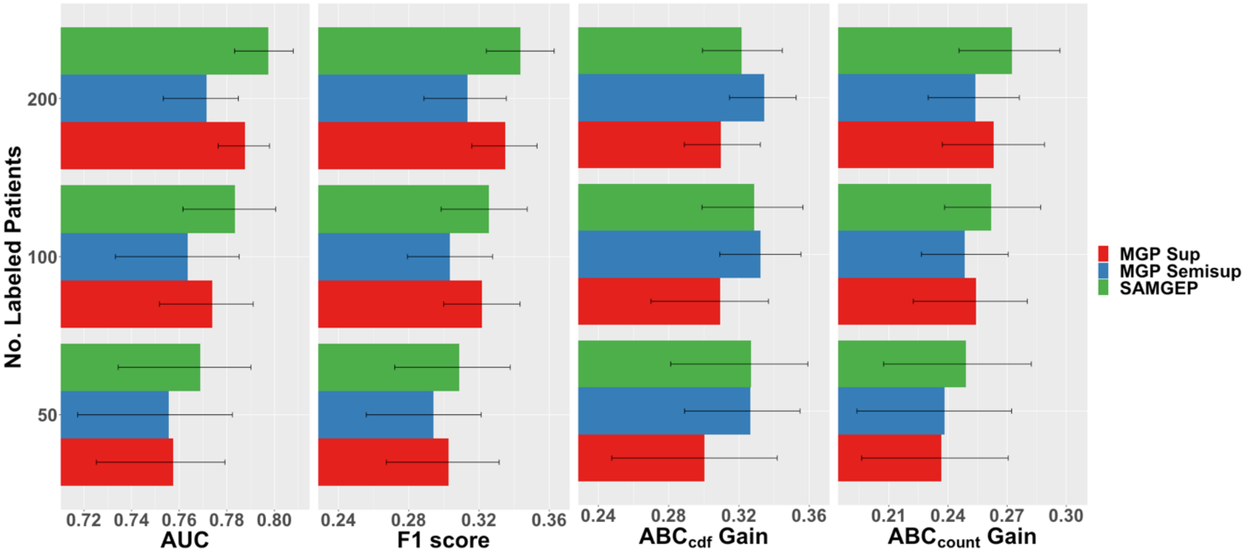

(B)

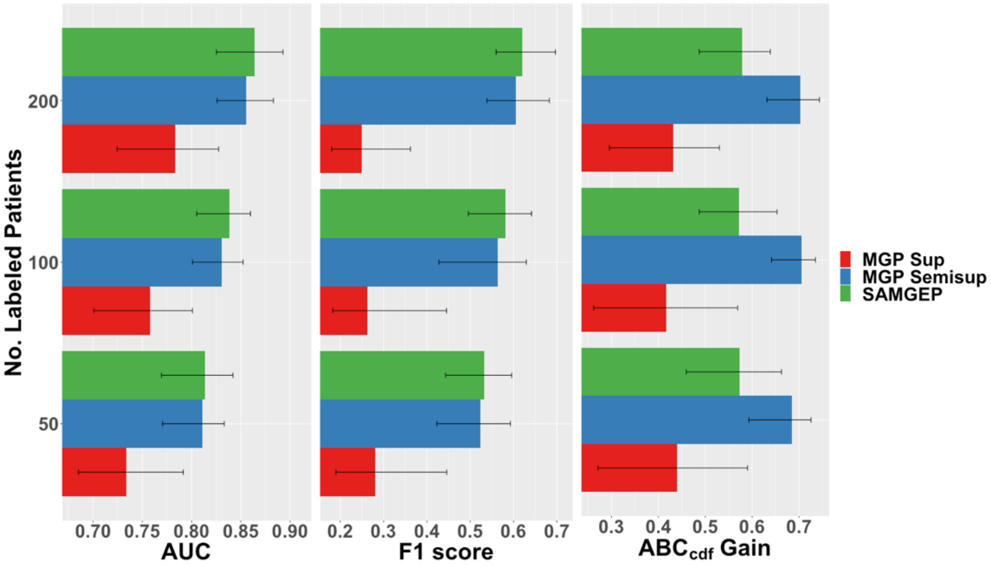

Figure S1: AUCs, F1 scores, ABC_{cdf} gains, and ABC_{count} gains for SAMGEP versus supervised and unsupervised MGP predicting MS relapse (top) and HF onset (bottom) using real-world EHR data. 95% confidence intervals were empirically estimated by bootstrapping with 100 replicates.

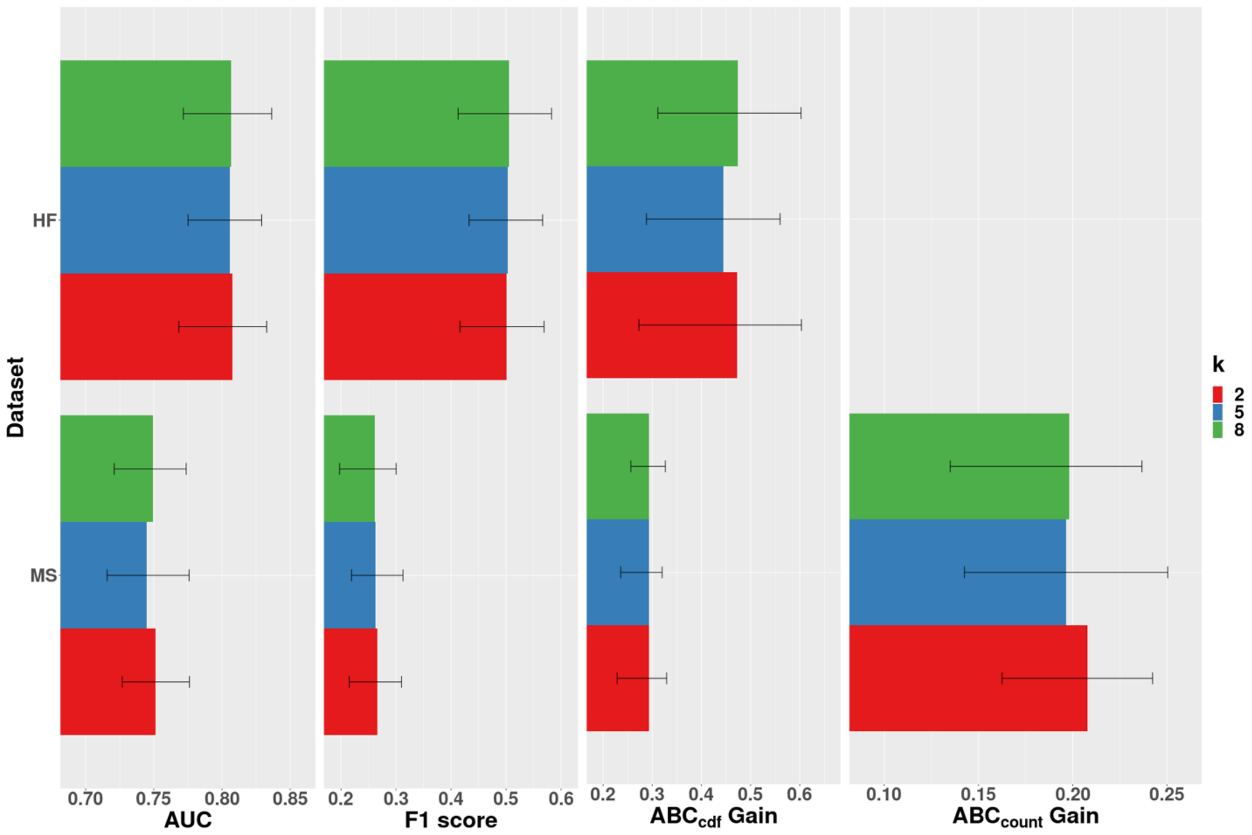

Figure S2: Sensitivity of SAMGEP to *k* in all *k*-fold cross-validation steps within the procedure. 95% confidence intervals were empirically estimated by bootstrapping with 100 replicates.

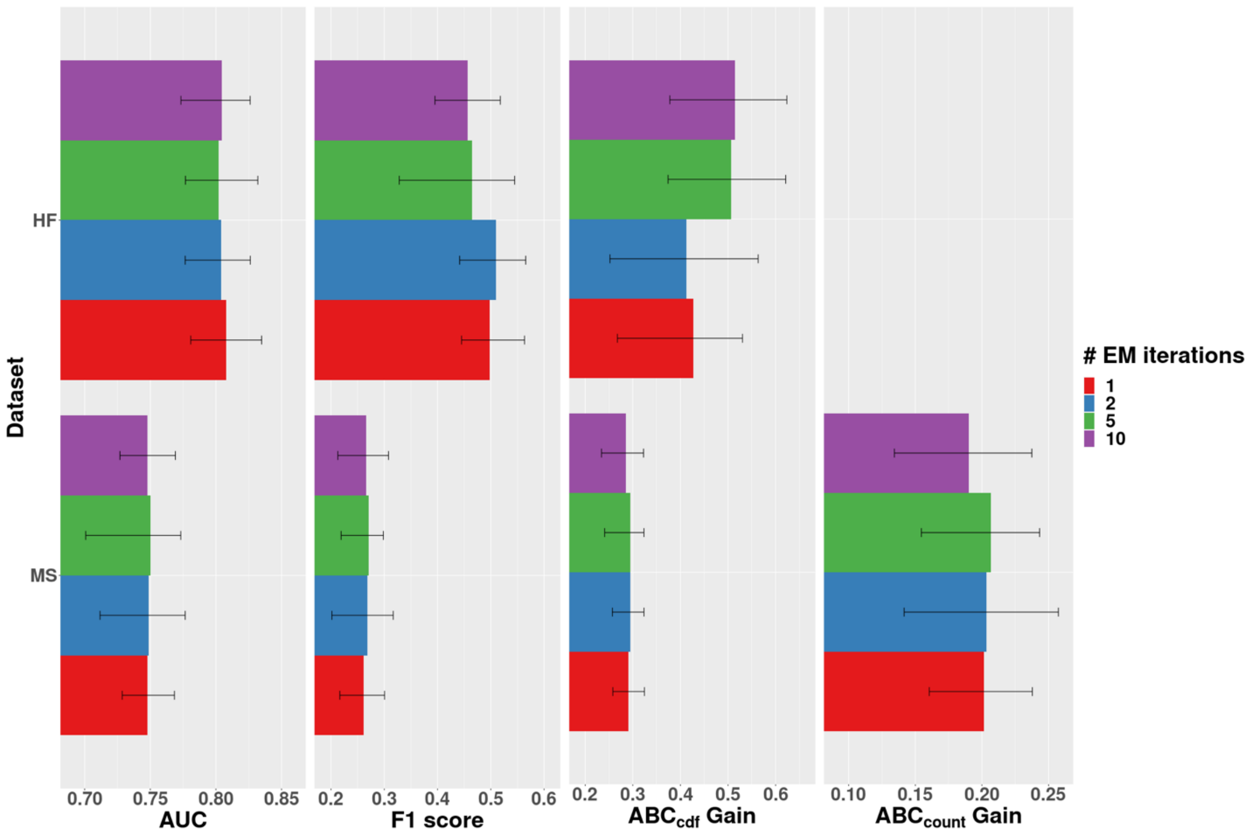

Figure S3: Sensitivity of SAMGEP to the maximum number of EM iterations allowed. 95% confidence intervals were empirically estimated by bootstrapping with 100 replicates.

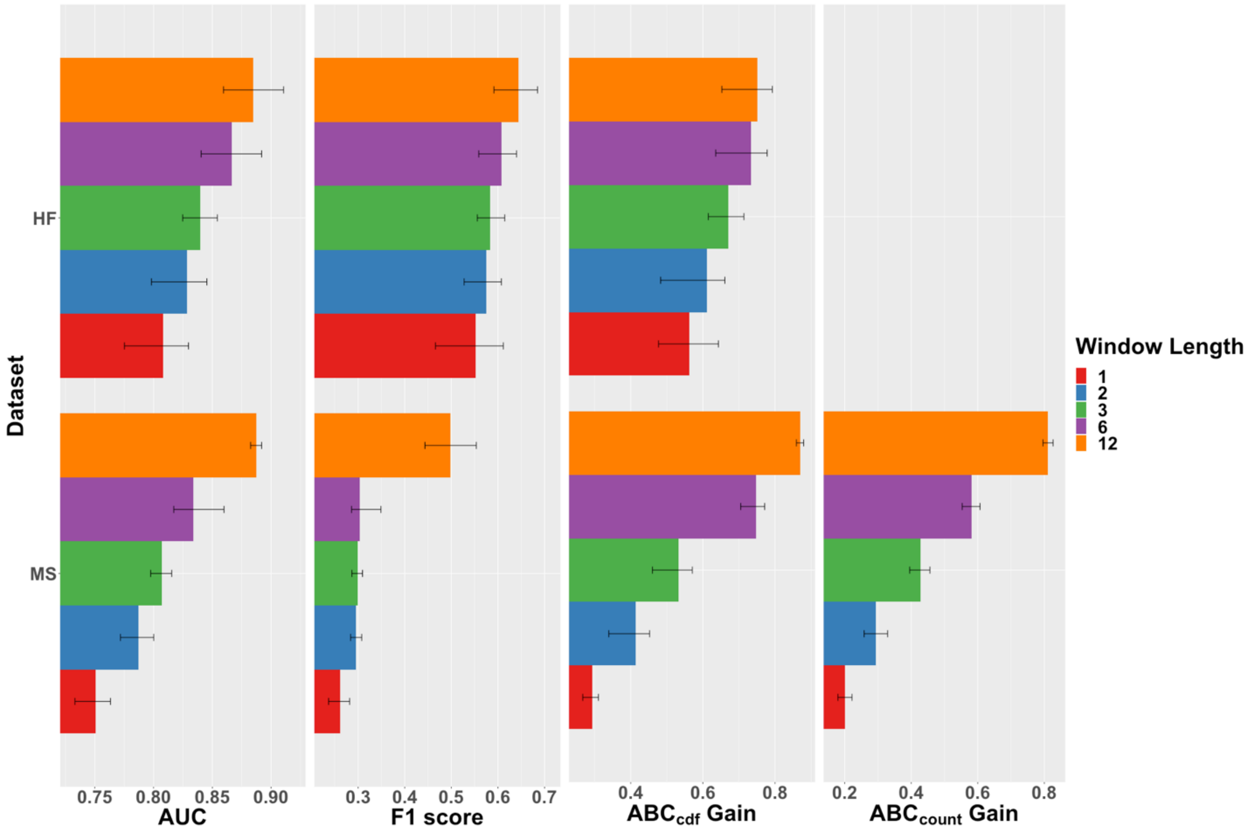

Figure S4: Sensitivity of SAMGEP to the window length used for data discretization. 95% confidence intervals were empirically estimated by bootstrapping with 100 replicates.

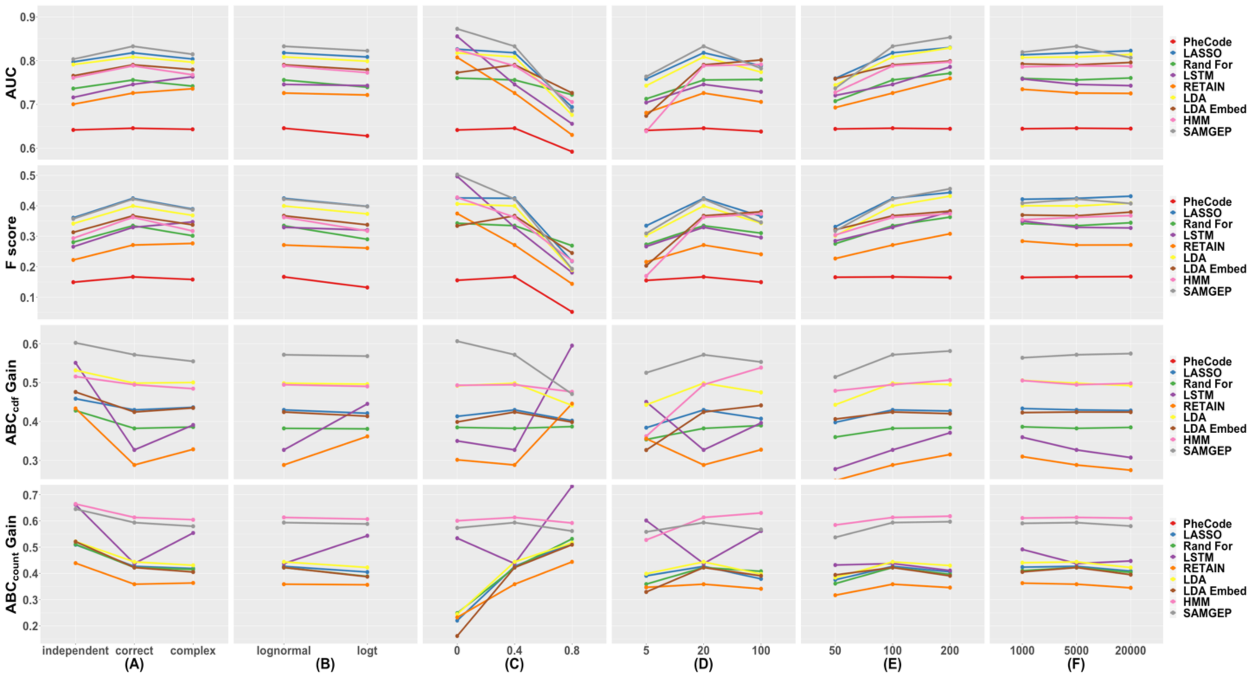

Figure S5: Robustness of SAMGEP and all comparator methods' AUCs, F scores, ABC_{cdf} gains, and ABC_{count} gains to various generative parameters, including the (A) specification of $Y|T$, (B) specification of $\boldsymbol{C}|Y$, (C) inter-temporal correlation parameter ρ, (D) number of informative (i.e. non-sparse) features, (E) number of labeled patients n, and (F) total number of patients N. Details of the experiments are delineated in the *Simulation Study* subsection of the Methods.

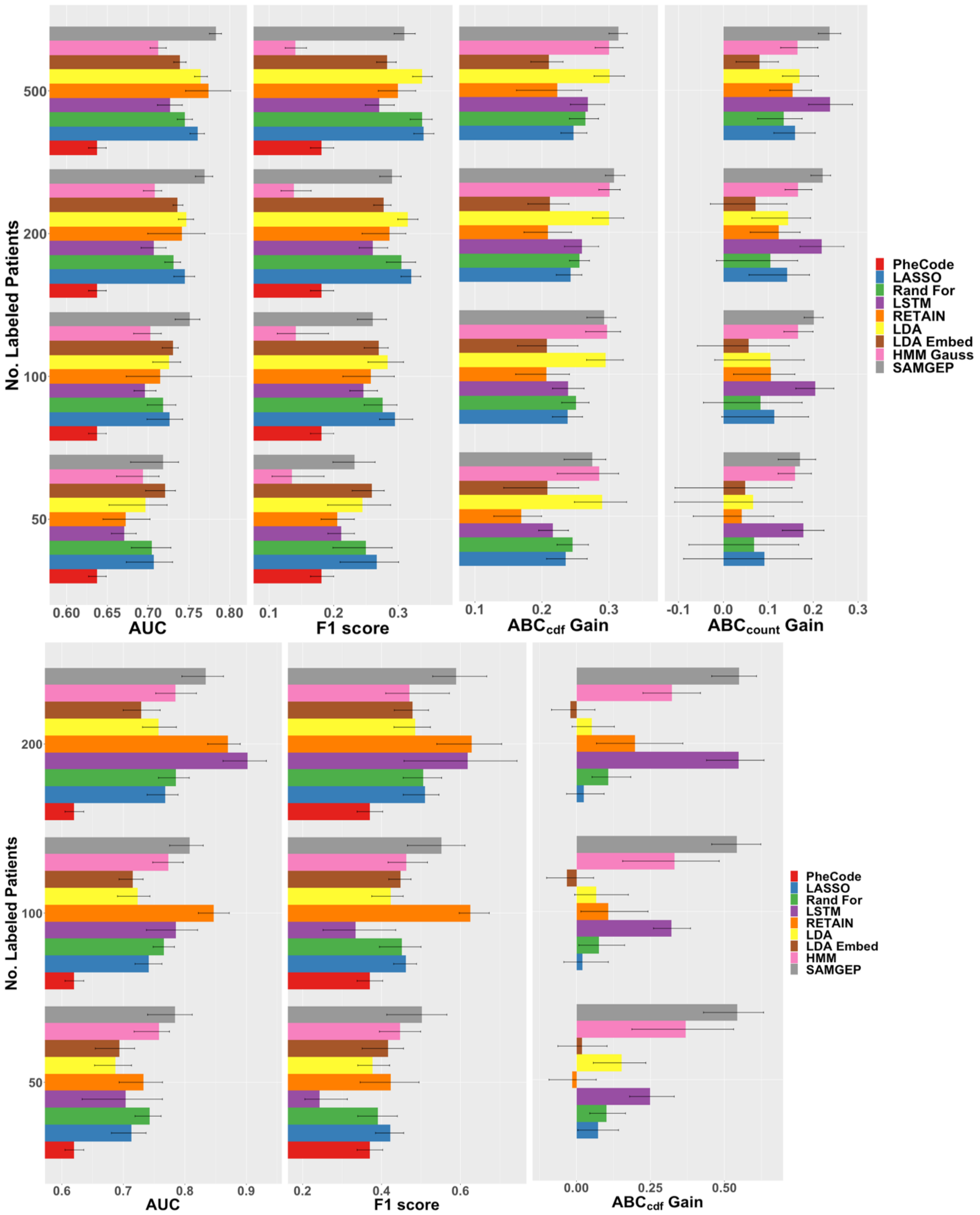

Figure S6: Predictive accuracies of SAMGEP and various comparator methods using real-world EHR data to predict (A) MS relapse with $n \in \{50,100,200,500\}$ labeled patients, and (B) HF onset with $n \in \{50,100,200\}$ labels. 95% confidence intervals were empirically estimated by bootstrapping with 100 replicates. See the *Evaluation Metrics* subsection of the Methods for details about the evaluation metrics.

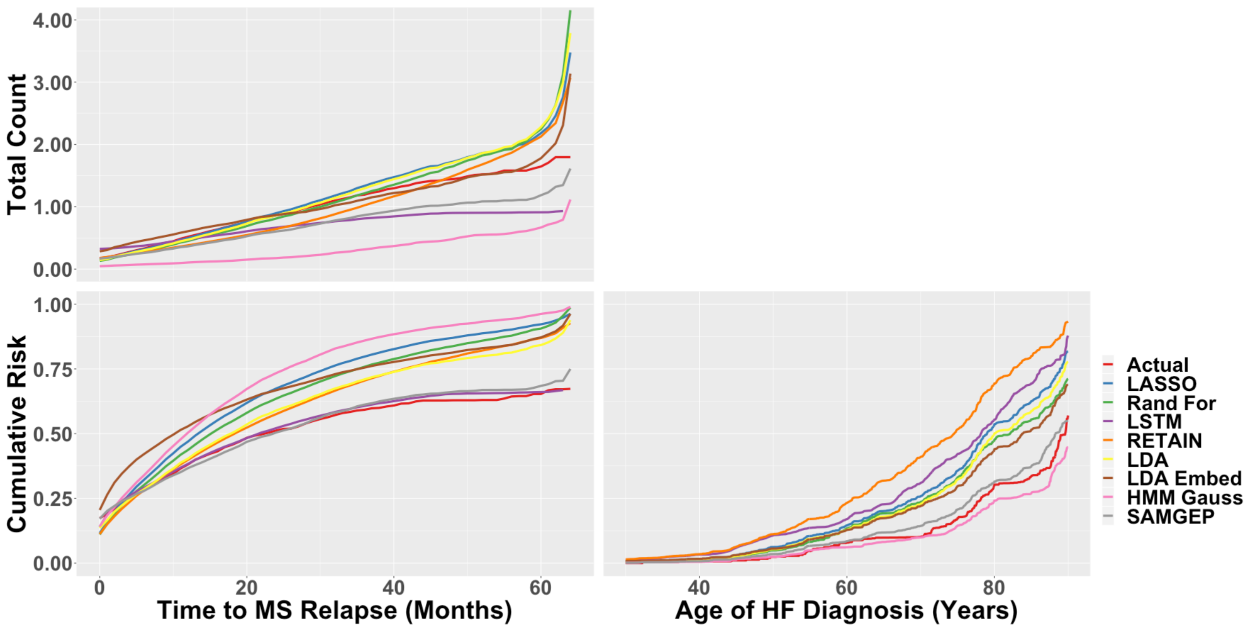

Figure S7: Estimation of population-wide cumulative probability (bottom) and counting process (top) curves for MS relapse (left) and HF development (right) using the identifications of SAMGEP and various comparator methods with $n = 100$ labeled patients.

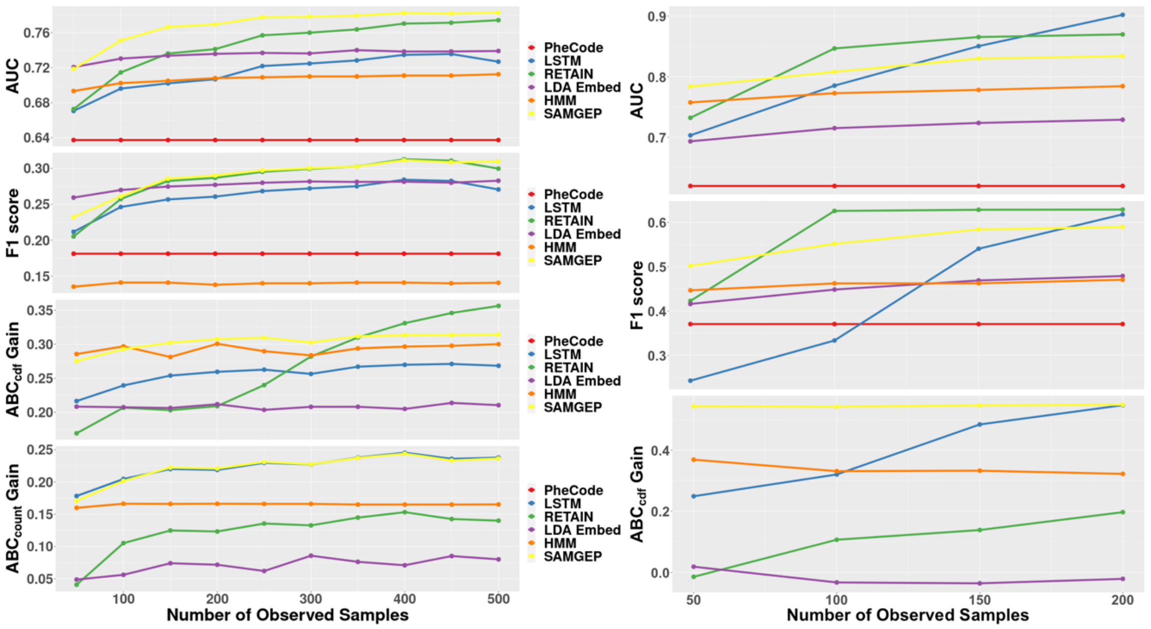

Figure S8: Line plots of predictive AUC, F1 score, ABC$_{cum}$, and ABC$_{count}$ over the number of observed labels *n* for SAMGEP and baseline comparators on the MS (left) and HF (right) datasets.

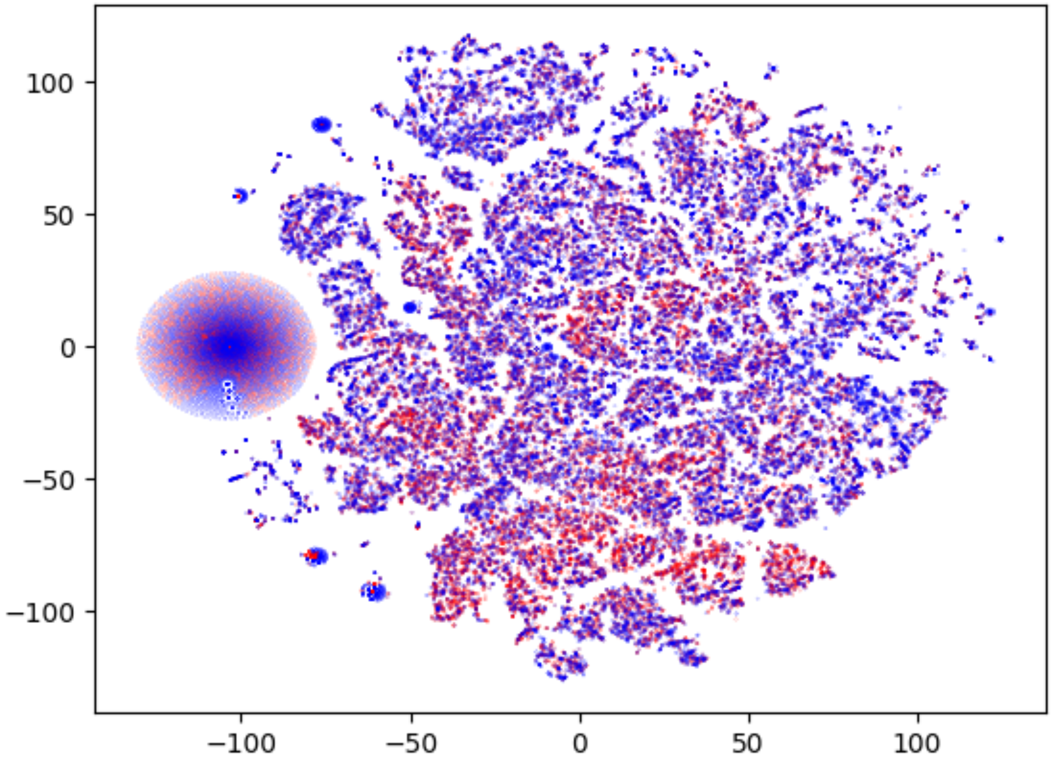

Figure S9: 2-dimensional t-SNE plot of the embedded MS dataset $\boldsymbol{X}$. Blue points represent CLIMB patients and red points represent non-CLIMB patients.
